# Scar Inhibition in Wound Healing: Mechanisms, Design, and Recent Advances

**DOI:** 10.1002/EXP.20240517

**Published:** 2026-02-17

**Authors:** Yong Kang, Yiwen Yang, Bin Yao, Zhuhong Zhang, Xiaoyuan Ji

**Affiliations:** ^1^ State Key Laboratory of Advanced Medical Materials and Devices Medical College, Tianjin University Tianjin China; ^2^ School of Pharmacy Key Laboratory of Molecular Pharmacology and Drug Evaluation Ministry of Education, Collaborative Innovation Center of Advanced Drug Delivery System and Biotech Drugs in Universities of Shandong, Yantai University Yantai China; ^3^ Department of Gastric Surgery Tianjin Medical University Cancer Institute & Hospital, National Clinical Research Center For Cancer, Tianjin Key Laboratory of Digestive Cancer, Tianjin's Clinical Research Center For Cancer Tianjin China; ^4^ Center For Nanomedicine Brigham and Women's Hospital, Harvard Medical School Boston Massachusetts USA

**Keywords:** biomaterials, immune modulation, scar inhibition, signaling pathways, wound healing

## Abstract

Scar inhibition plays a crucial role in wound healing, particularly in the prevention of excessive scar formation during skin repair. While scar formation is a natural part of the healing process, improper scarring can lead to functional impairment, cosmetic defects, and psychological impacts. Therefore, understanding the mechanisms behind scar inhibition and exploring therapeutic strategies is essential for improving clinical outcomes. This review summarizes the primary mechanisms of scar formation and inhibition, including the regulation of collagen deposition, modulation of the inflammatory response, and control of cell proliferation and migration. In recent years, novel therapeutic approaches have emerged for scar inhibition, including gene therapy, stem cell treatments, and localized drug delivery systems, and the use of biomaterials. These methods not only enhance the effectiveness of scar treatment but also improve the biocompatibility and durability of the healing process. Although some of these approaches have shown promising results in early‐stage studies, challenges remain for clinical applications, such as the individualization of treatment plans and the sustainability of outcomes. Finally, this review discusses future research directions and proposes strategies to enhance the potential of scar inhibition therapies and their clinical translation.

## Graphical abstract

This review elaborates on current research on immune modulation in tissue regeneration, emphasizing the roles of proinflammatory and anti‐inflammatory cells. This review also explored the potential of biomaterials for modulating the wound environment to promote scarless healing. Advanced strategies are discussed for their potential to functional skin repair.

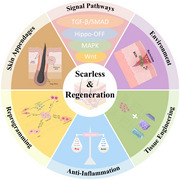

## 1 Introduction

Scar, a ubiquitous consequence of wound healing and surgical procedures, can have impacts that extend beyond the physical realm, causing emotional distress and functional limitations, and significantly reducing the quality of life. Hypertrophic scars and keloids, in particular, pose considerable challenges due to their tendency to extend beyond the original wound boundaries. Consequently, there is a burgeoning interest in devising effective strategies for the prevention and management of excessive scarring. In recent years, significant strides have been made in elucidating the underlying mechanisms of scar formation [1]. Multiple factors, including inflammation, fibroblast proliferation, and collagen deposition, contribute substantially to scar development. These insights have catalyzed the exploration of novel therapeutic modalities aimed at alleviating scar formation. From conventional topical treatments to cutting‐edge surgical interventions, various approaches are being investigated to reduce scars and promote wound healing [2].

Inflammation serves as a cornerstone in the cascade of events leading to scar formation [3]. Following tissue injury, an inflammatory response involving the release of various cytokines and growth factors is initiated. These signaling molecules recruit inflammatory cells to the site of injury, where they orchestrate tissue repair processes. However, dysregulated inflammation can contribute to excessive scar formation. Therefore, strategies aimed at modulating the inflammatory response represent a promising avenue for scar prevention. Fibroblasts, the principal cellular mediators of wound healing, play a pivotal role in scar formation. Upon activation, fibroblasts proliferate and migrate to the wound site, where they synthesize and remodel the extracellular matrix (ECM) [4]. However, aberrant fibroblast activity can lead to excessive ECM deposition, resulting in the formation of hypertrophic scars and keloids. Regulating the activation and behavior of fibroblasts is an attractive approach for scar modulation [5]. Collagen, the hallmark structural protein of connective tissues, undergoes dynamic remodeling during wound healing. In normal wound healing, collagen fibers are organized in a parallel orientation, facilitating tissue strength and elasticity. Conversely, in hypertrophic scars and keloids, collagen fibers are arranged haphazardly, leading to the characteristic raised and firm appearance of these scars. Therapeutic interventions aimed at promoting proper collagen alignment and remodeling hold promise for improving scar aesthetics and functionality.

Several therapeutic modalities have been explored for the prevention and treatment of excessive scarring. Topical medication treatments, such as silicone gels [6] and sheets, are widely used to manage hypertrophic scars and keloids. These interventions function by occluding the scar site, hydrating the skin, and modulating collagen synthesis. Silicone gel sheets are non‐invasive topical treatments widely utilized for various types of scars, including hypertrophic and keloid scars. They are user‐friendly, generally well‐tolerated, and associated with high patient compliance and safety, exhibiting minimal side effects. However, the efficacy of silicone gel sheets is contingent upon consistent and prolonged application, typically requiring daily use for several months to achieve optimal results. Their effectiveness is limited, particularly concerning older scars, where they may yield suboptimal outcomes. Additionally, corticosteroid injections are frequently employed to reduce inflammation and inhibit fibroblast activity in hypertrophic scars and keloids [7]. This injectable medication effectively inhibits the proliferation of myofibroblasts and reduces collagen deposition. However, it is associated with notable side effects, including skin atrophy and pigmentary changes. Moreover, due to its intralesional administration, the treatment must be performed by trained professionals, which limits the convenience and accessibility of its use. Physical therapies, including laser therapy and cryotherapy, are commonly employed in scar management. Laser therapy improves scar appearance by utilizing selective photothermolysis to target abnormal vasculature, reduce inflammation, and promote collagen remodeling. Clinically, pulsed dye laser (PDL) and carbon dioxide (CO_2_) lasers are frequently used to treat hypertrophic scars and keloids. However, laser therapy is associated with high treatment costs, requires multiple sessions, and carries the risk of post‐treatment complications such as hyperpigmentation or hypopigmentation, necessitating careful postoperative care. Cryotherapy, on the other hand, destroys scar tissue by inducing cellular damage through extreme cold, leading to necrosis and subsequent sloughing of the scar, thereby facilitating the regeneration of normal tissue. It is a simple and effective modality, particularly for small keloids, and can be used in combination with other treatments. Nevertheless, cryotherapy is often accompanied by considerable pain during treatment and may cause local blistering and pigmentary alterations. These minimally invasive procedures offer the advantage of targeted scar treatment with a reduced risk of adverse effects. For large scar tissues that impair function, surgical excision may be considered a viable treatment option. However, due to the high recurrence rate of keloids, adjuvant therapies such as radiation therapy, pressure therapy, or intralesional pharmacologic injections are often required postoperatively to minimize the risk of recurrence. To ensure both safety and therapeutic efficacy, tissue engineering strategies that integrate biologically active cells or cell‐derived components with biomaterials have emerged as promising and clinically relevant approaches for scar treatment. Tissue engineering approaches aim to reconstruct damaged tissue using biomimetic scaffolds and cell‐based therapies [8]. By mimicking the native tissue microenvironment, these scaffolds provide structural support and promote cell infiltration and differentiation. Additionally, cell‐based therapies, such as mesenchymal stem cell transplantation [9], have the potential to modulate the inflammatory response and enhance tissue regeneration. While still in the experimental stages, tissue engineering holds immense promise for scar management by enabling the regeneration of functional tissue with minimal scarring.

In this review, we aim to provide a comprehensive overview of the current understanding of scar formation, highlighting recent advancements in the field of scar suppression. By examining the mechanisms underlying scar formation and evaluating the efficacy of different treatment modalities, we aimed to offer insights into the future direction of scar management. Ultimately, our goal is to contribute to the development of improved strategies for inhibiting scars and enhancing the well‐being of individuals affected by excessive scarring.

## 2 Scar‐Forming Mechanisms

### 2.1 Wound Healing and Scar Formation

Wound healing is a complex process involving the synergistic action of different skin component cells, recruited immune cells, and the formation of an ECM. It is divided into four stages: hemostasis, inflammation, proliferation, and remodeling, with scar formation occurring during the remodeling stage. Wounding causes local vasoconstriction, platelet aggregation, and initial hemostasis, which activates the coagulation cascade, leading to the formation of a fibrin clot and subsequent hemostasis [10].

The next stage is the inflammatory stage, which varies in duration depending on the size and severity of the wound and generally lasts 4–6 days. In the wound area, inflammatory cells are recruited through chemokine signaling pathways from the immune system, resulting in a sustained influx of lymphocytes, neutrophils, and macrophages. Neutrophils play a vital role in defending against invading microorganisms and clearing pathogens and tissue debris in wounds by releasing nitrogen and reactive oxygen species (ROS) [11]. Neutrophils also play a role in recruiting other inflammatory cells, such as monocytes derived from macrophages. Mature macrophages are formed under the influence of interferon‐γ (IFN‐γ) and bacterial products and can clear residual tissue debris and microorganisms in wounds. M1‐type macrophages are considered inflammatory [12]. After complete clearance, most M1 macrophages undergo apoptosis, and the remaining M1 macrophages transform into M2 macrophages, thus entering the proliferation stage [13].

Fibroblasts are massively recruited and rapidly proliferate and differentiate during the proliferation phase, where they produce fibronectin, collagen, and other ECM components at the wound site to promote wound healing [14]. Under the influence of various cell growth factors, such as transforming growth factor‐β (TGF‐β) and fibroblast growth factor (FGF), fibroblasts and endothelial cells continue to proliferate, migrate, and differentiate, promoting the formation of new blood vessels and granulation tissue in the dermis. Moreover, the wound edge will continue to contract due to the continuous generation of myofibroblasts with contractile functions. Myofibroblasts are mainly derived from resident fibroblasts and are the key medium for fibrotic tissue remodeling [15]. Myofibroblasts can deposit more ECM and have stronger contractile force to help the wound contract and shrink [16]. New granulation tissue appears in the dermis and below, and the epidermis undergoes re‐epithelialization.

**FIGURE 1:**
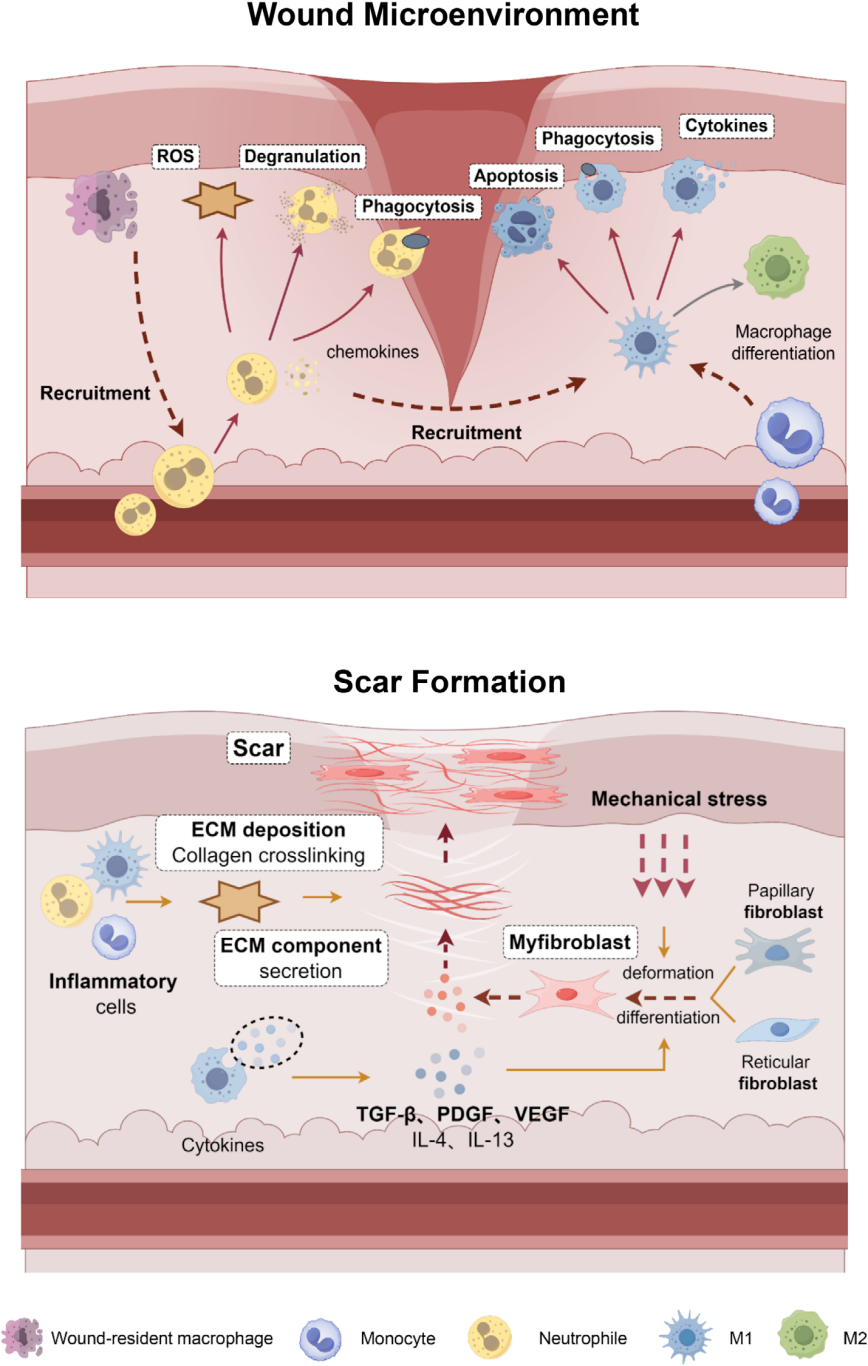
Scar formation process and mechanism.

Subsequently, the wound enters the final remodeling stage, where the main event is the degradation and remodeling of the ECM deposited in the previous stage. Granulation tissue gradually becomes scar tissue, and this stage can last 4–26 days or even longer. In most previous stages, endothelial cells, fibroblasts, and immune cells undergo apoptosis. Under the action of collagenases and matrix metalloproteinases (MMPs), the protein components in the formed ECM are degraded, type III collagen is partially replaced by type I collagen, collagen fiber cross‐linking increases, and the reshaping of new blood vessels and skin appendages is basically completed. The soft pink granulation tissue at the wound site gradually hardens and darkens to form a scar (Figure 1) [17, 18].

### 2.2 The Role and Drawbacks of Scars

Scars are the product of the natural repair response of the body's wounds, protecting the wound from exposure to the external bacterial environment and providing a closed space for internal skin reshaping. The molecular mechanism of scar formation involves the interaction of various cytokines, cell types, and molecular signaling pathways. Inflammatory mediators play a key role in the early stages of scar formation. During inflammation, damaged tissues release a large number of cytokines, such as tumor necrosis factor‐α (TNF‐α), transforming growth factor‐β (TGF‐β), interleukin‐6 (IL‐6), and interleukin‐1 (IL‐1), promoting fibroblast proliferation and collagen synthesis, thus leading to scar formation. Among these pathways, the TGF‐β/Smad pathway is a typical signaling pathway involved in scar formation. The binding of TGF‐β to its receptor leads to the activation and phosphorylation of Smad proteins to promote fibroblast transformation into myofibroblasts. The activation of Smad proteins can also promote collagen synthesis, increasing the quantity and density of collagen fibers in scar tissue and leading to the thickening and hardening of scar tissue.

The essence of a scar is a tissue that does not have the normal tissue structure and physiological function of the skin, lacks normal tissue vitality, and is an incomplete tissue. It not only destroys the beauty of the skin but also hinders the physiological function of related tissues or organs and may even lead to deformity. Hypertrophic scarring (HS) is a skin disease characterized by excessive fibrosis of the skin. They are more likely to occur after burns and surgeries. After skin injury heals, the scar continues to proliferate and can develop into hypertrophic scars [19]. Hypertrophic scars not only seriously affect patients’ appearance but also cause psychological damage to patients. Moreover, these patients may further progress to scar cancer. Scar cancer is more likely to occur in contracture scars caused by burns, often occurring near joint areas. The fragility of scar tissue and the continuous destruction caused by joint activity eventually lead to cancer. Therefore, it is necessary to fully understand the mechanism of scar formation and reduce scar formation after trauma, both for human health and to meet people's demands for skin beauty [20] (See Table 1).

**FIGURE 2:**
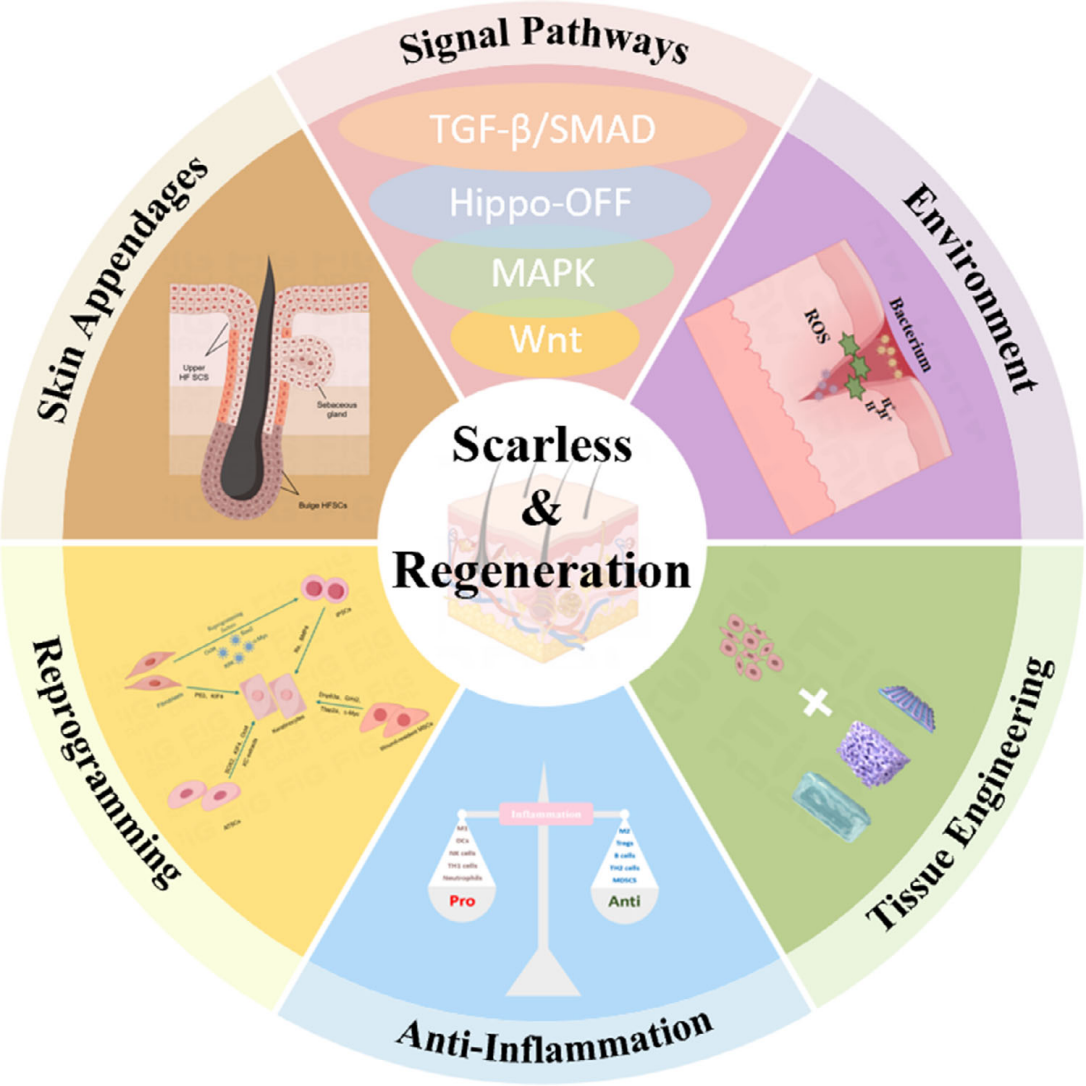
Key factors and methods to reduce scarring.

Current treatment strategies for mature scars can be divided into two main categories. On the one hand, after wound healing is completed and the scar is fully formed, surgical or laser excision is used, that is, invasive treatment [21]. After the scar tissue matures, the surrounding tissue can no longer generate new dermal tissue, and these methods are mainly used for the removal of hypertrophic scars that cause significant changes in the patient's life. On the other hand, during the wound healing process, wet dressings are applied to maintain the wound healing process in a moist environment, that is, moist healing. This method can make the scar produced after wound healing shallower, and the skin at the trauma site is more aesthetically pleasing. However, at the same time, moist healing slows the speed of wound healing; it only plays a role in surface aesthetics, and the functional components of damaged skin are not restored [22]. Therefore, research on treatment strategies that restore both the beauty and functionality of the skin is ongoing. This article will introduce the current research progress on scarless wound healing from different causes of scar formation (Figure 2).

## 3 Current Research on Reducing Scar Formation

**TABLE 1:** Summary of current clinical and investigational therapies for scar reduction.

Therapeutic category	Drug/Treatment method	Mechanism of action	Research or approval status
Pharmacotherapy	Triamcinolone, dexamethasone tazarotene	Inhibits pro‐inflammatory cytokines and fibroblast activation; reduces collagen deposition	Widely used in clinical practice via intralesional injection
	Fresolimumab SB‐431542	Inhibit TGF‐β/Smad signaling	At the preclinical research stage
Topical dressings	Silicone dressings, silicone gel sheets	Forms a semi‐permeable membrane to retain moisture and reduce mechanical tension	FDA‐approved, widely applied clinically
Physical therapy	Pulsed dye laser (PDL), CO_2_ laser	Destroys abnormal vasculature and promotes collagen remodeling	Multiple clinical applications, some with trial registration
	Pressure therapy	Provide sustained external compression to the wound, reducing capillary perfusion and local oxygen tension	Widely used in clinical practice
Biologics	Etanercept, Infliximab	Inhibits TNF‐α signaling and modulates immune response	Small‐scale studies, early‐phase clinical trials
Stem cell therapy	MSCs/iPSCs‐derived exosomes	Paracrine regulation of immune response, angiogenesis, and ECM remodeling	Preclinical or phase I studies
Gene editing technology	CRISPR‐Cas9 system	Targets TGF‐β, Wnt, and related fibrotic signaling pathways	At preclinical research stage

### 3.1 Immune Modulation

The immune system plays a key role in tissue repair and largely determines the degree of final scar formation. Moreover, the regenerative potential of an individual's tissue is positively correlated with their immune system capabilities. Therefore, to achieve complete regeneration of skin tissue, research on controlling immune responses in the most orderly manner is indispensable. Wound repair immune cells can be divided into two categories: proinflammatory and anti‐inflammatory cells.

During the inflammatory stage, a large number of immune cells, including macrophages, neutrophils, monocytes, and different T‐cell subsets, are recruited to the wound site and perform different tasks. These immune cells collectively regulate the orderly occurrence of tissue regeneration [23]. When trauma occurs, M1 macrophages first respond to damage, and under the attraction of chemokines, TGF‐β, and small molecules produced by bacteria at the wound site, neutrophils, lymphocytes, and various inflammatory cells continuously infiltrate the wound. Monocytes are attracted to the wound site by PDGF and TGF‐β, as well as by the breakdown products of elastin and collagen. In this stage, the role of these inflammatory cells is to clear residual tissue debris, pathogens, apoptotic cells, and bacteria at the wound site, preparing the ground for subsequent tissue regeneration. Neutrophils play an important role in resisting invading microorganisms and recruiting other inflammatory cells [11].

After inflammatory cells complete the task of clearing the wound, they gradually undergo apoptosis, and anti‐inflammatory cells begin to be massively recruited to reduce the inflammatory response, which is beneficial for the formation of new blood vessels, endothelial cells, and fibroblasts, among other skin component cells, to rapidly proliferate and differentiate. Specifically, M1 macrophages gradually undergo apoptosis, and the remaining few M1 macrophages and monocytes transform into M2 anti‐inflammatory macrophages [13]. M2 macrophages not only reduce inflammation but also release various important cell growth factors, such as TGF‐β and platelet‐derived growth factor (PDGF), which activate fibroblasts and promote their proliferation, thereby reshaping the ECM, enhancing collagen deposition, and facilitating skin fibrosis. Other key anti‐inflammatory cells include regulatory T cells (Tregs), T helper 2 (TH2) cells, and γδ T cells. Numerous studies have shown that in scarless wound healing, Tregs and TH2 immune cells exhibit increased activity and upregulated gene expression, contributing to a regenerative immune environment [24]. These findings have prompted growing interest in harnessing anti‐inflammatory immune responses to guide wound repair. One emerging strategy involves the use of immunomodulatory biomaterials to direct the local immune milieu toward a regenerative phenotype. By designing biomaterials with tailored physicochemical properties and implanting them at the wound site, researchers aim to elicit a favorable foreign body response that recruits specific immune cell subsets conducive to tissue regeneration.

**FIGURE 3:**
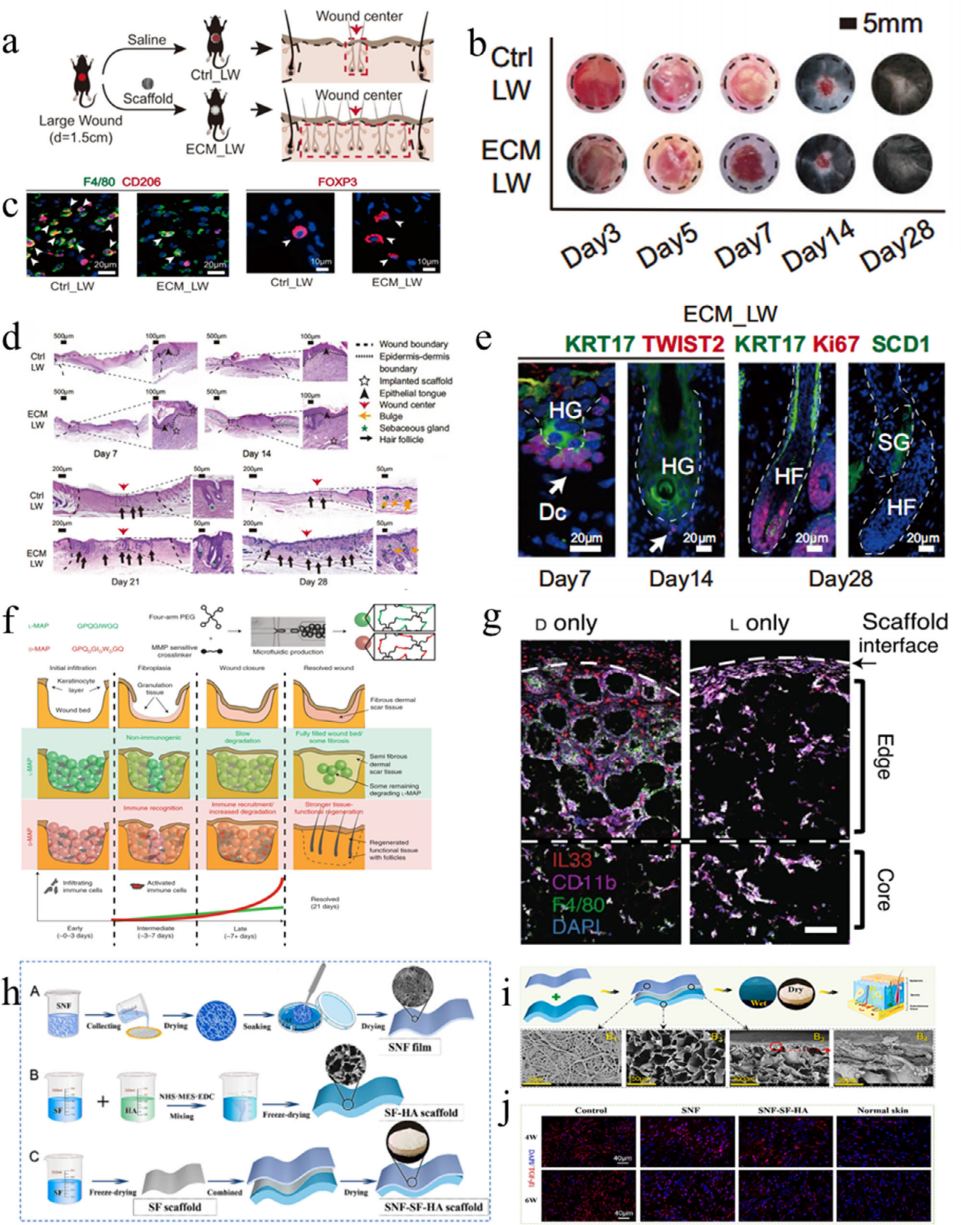
Strategies for promoting scarless wound healing by modulating the immune microenvironment. (a) The process of creating a skin wound model. (b) Wound area recording. (c) IF images of AIM2 (F4/80 + CD206+) and Treg2 (FOXP3+) staining. (d) H&E images. (e) IF images of HFs stained for KRT17 (green), TWIST2 (red), Ki67 (red), and SCD1 (green) [25]. Copyright 2023, Springer Nature. (f) Preparation and degradation records of hydrogels. (g) High‐resolution confocal immunofluorescence imaging of CD11b, F4/80, DAPI, and IL‐33 [28]. Copyright 2021, Springer Nature. (h) Preparation process of the SNF‐SF‐HA scaffold. (i) Preparation process of the SNF‐SF‐HA scaffold and SEM. (j) Immunofluorescence of TGF‐β1 in different groups [29]. Copyright 2023, Elsevier.

Building on this concept, a recent study in mice demonstrated that implantation of a 3D‐printed ECM scaffold into full‐thickness skin wounds can effectively stimulate the adaptive immune system. Histological analysis of the wound center revealed that the regenerated skin tissue exhibited enhanced hair follicle formation and significantly reduced scarring after ECM scaffold implantation (Figure 3a,b) [25]. Immunological assessments further indicated a marked increase in Tregs and TH2 cells in the ECM‐treated group compared to controls, underscoring the immunoregulatory advantage conferred by the scaffold and its potential to promote scarless tissue regeneration. Tregs, which are typically located in the dermis near the hair follicles (HFs) of normal skin, are known to maintain immune homeostasis equilibrium. It has been reported that Tregs can inhibit excessive type 2 inflammatory responses through the expression of Gata2, preventing excessive tissue fibrosis, that is, preventing excessive deposition of ECM and collagen on M2 macrophages (Figure 3c–e). Clinical IL‐2 treatment (which promotes Treg proliferation and activation) has also been proven to effectively reduce chronic skin fibrosis [26]. Moreover, Tregs can promote the differentiation of hair follicle stem cells (HFSCs) through the Jag1‐Notch1 pathway, promoting hair follicle HF regeneration [27]. In addition, by analyzing the immune microenvironment of the wound site, it was found that after the implantation of this ECM scaffold, the proportion of neutrophils in the Ctrl‐LW group was greater, and the expression of proinflammatory genes was greater, while there was no significant difference in DC cells between the two groups. Interestingly, spatial gene expression profiling of wound site cells revealed that the ECM‐LW group had an enrichment of DC cells in the dermis in the early stage, which is consistent with the early characteristics of hair follicle formation. Furthermore, Donald R. Griffin's team modified MAP gels to reduce the in vivo degradation rate of hydrogels to maintain a high porosity for a longer period [28], supporting cell growth and surrounding tissue infiltration. Although the gel degraded quickly, the MAP gel itself immediately activated the adaptive immune induction of skin regeneration, which was achieved by recruiting IL‐33^+^ II‐type myeloid cells through the hydrogel scaffold (Figure 3f,g). These studies show that biomaterials have the ability to reshape the spatial heterogeneity of the wound environment and activate the adaptive immune system to maintain an advantageous state, which is highly important for the design of biomaterials aimed at achieving scarless skin regeneration through immune modulation.

Furthermore, combining cells with strong regenerative potential with biomaterials can further promote the functional regeneration of deficient tissues, combining biology and materials for better results. An artificial skin bilayer prepared by electrospinning, with a silk fibroin nanofiber membrane (SNF) as the epidermis and a silk (SF)/hyaluronic acid (HA) scaffold as the dermis layer, simulates the structure of human normal skin, and human umbilical vein endothelial cells are planted in the bilayer scaffold (Figures 3h–j). Experiments have shown good cell viability, indicating that the designed biomaterial scaffold has good biocompatibility [29]. The scaffold was transplanted into rabbit ear wounds and showed good anti‐inflammatory and scar inhibition effects. Due to the presence of HA and SF, the scaffold has high water absorption and swelling properties, promoting the absorption of tissue fluid and maintaining the humidity of the wound microenvironment. The porous structure of the scaffold also provides connectivity for the transport of nutrients and oxygen and supports cell adhesion and growth [30]. Similarly, bilayer scaffolds are also designed by imitating the components of the ECM, indicating that ECM biomaterials have promising application prospects in scarless skin regeneration and functional restoration.

**FIGURE 4:**
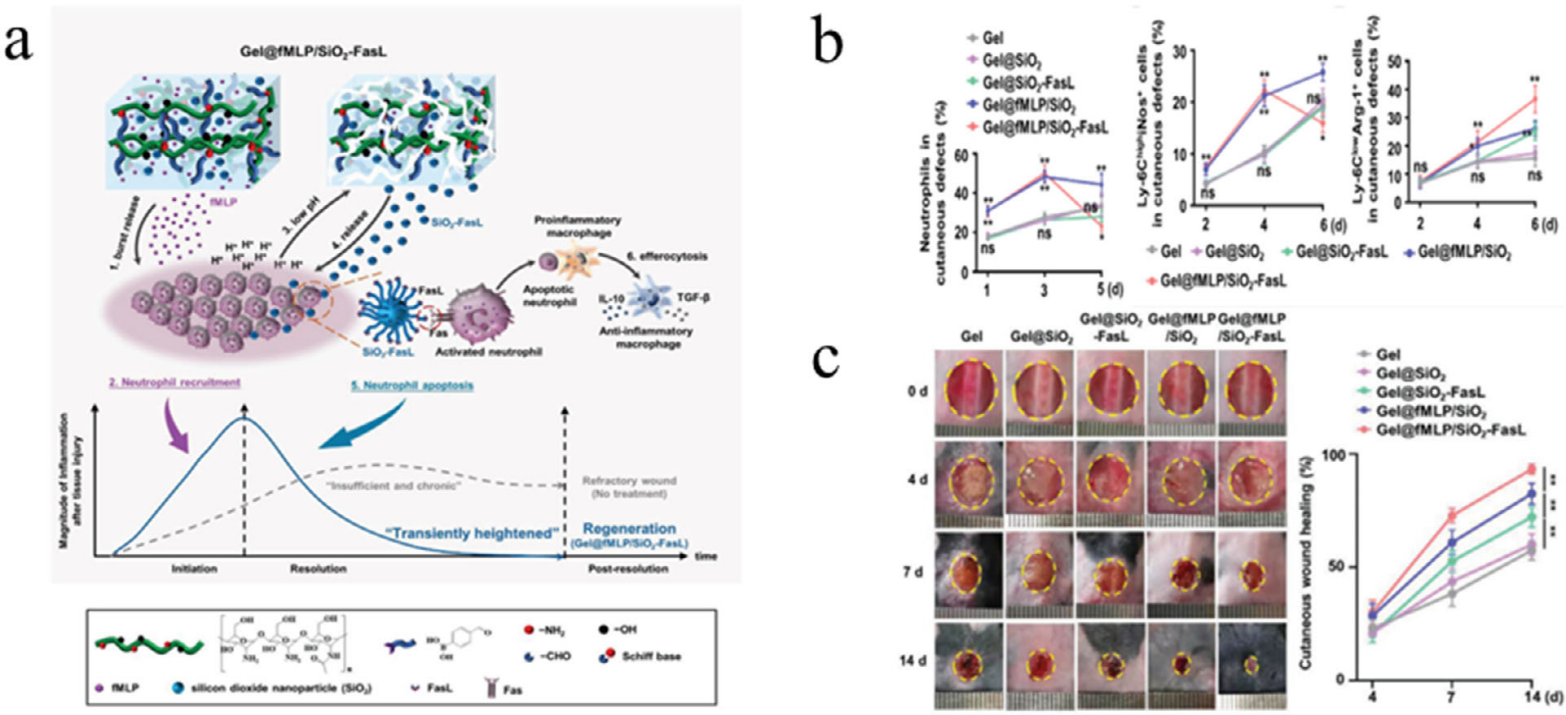
Strategies for promoting wound healing through inflammatory responses. (a) Schematic diagram of Gel@fMLP/SiO2‐FasL material preparation. (b) Quantitative analysis of neutrophils in wounds. (c) Photographic documentation of wound healing [31]. Copyright 2022, Wiley‐VCH.

However, not only can reducing inflammation promote wound healing, but the inflammatory response also plays an indispensable role in wound healing. A mixed biomaterial that induces a temporary high inflammatory response to accelerate chronic wound healing [31] formyl‐Met‐Leu‐Phe (fMLP) and FasL coupled to silica nanoparticles (SiO_2_‐FasL) was added to a pH‐responsive hydrogel matrix (Figure 4a). The release of fMLP leads to the recruitment of a large number of neutrophils, causing a temporary inflammatory response. At this time, a large number of neutrophils will reduce the wound pH, causing the hydrogel to degrade and expose the contained SiO_2_‐FasL. Through FasL‐Fas signaling, activated neutrophils are induced to undergo apoptosis, and the inflammatory response is triggered in a timely manner. When macrophages clear excess neutrophils, this clearance process promotes the polarization of M1 macrophages to the M2 phenotype, thus accelerating the wound healing process and promoting tissue regeneration (Figure 4b,c) [32, 33]. This mixed biomaterial can achieve reprogramming control of the inflammatory response process, and unlike most studies aimed at reducing inflammation, the induction of severe inflammation provides a new perspective for wound healing research.

Current clinical approaches aimed at modulating immune responses to promote wound healing and reduce scar formation primarily focus on controlling inflammation, regulating immune cell activity, and facilitating the transition of the immune microenvironment toward a reparative phenotype. These strategies encompass multiple levels, including pharmacological agents, biomaterials, and cell‐based therapies. For example, corticosteroids such as triamcinolone acetonide and dexamethasone are widely used for intralesional injection in hypertrophic scars and keloids. They function by suppressing the expression of pro‐inflammatory cytokines (e.g., TNF‐α, IL‐1β, and IL‐6), reducing immune cell infiltration, and inhibiting fibroblast activation and excessive collagen deposition. Additionally, certain biologic agents like Etanercept and Infliximab, originally developed for chronic inflammatory conditions such as rheumatoid arthritis and psoriasis, have shown promise in small‐scale studies for the management of chronic wounds, particularly in controlling local inflammation. Given the complexity and dynamic nature of immune responses in the wound microenvironment, immune modulation has emerged as a critical focus in tissue engineering and regenerative medicine. However, therapeutic strategies that center on immune microenvironment modulation remain largely auxiliary or at early developmental stages. Clinical implementation still faces numerous challenges. Many immunomodulatory interventions, including exogenous immune cells, immunosuppressive agents, and cytokine therapies pose risks of systemic immune interference, potentially leading to infection, tumorigenesis, or immune dysregulation. Furthermore, due to the highly individualized nature of immune responses, treatment efficacy can vary significantly among patients, influenced by factors such as age, metabolic status, diabetes, and other comorbidities. Although immunomodulation holds considerable promise for wound healing, its clinical translation is currently limited by concerns regarding safety, cost‐effectiveness, regulatory complexity, and technical execution. Multidisciplinary collaboration, standardized protocols, streamlined manufacturing processes, and sustainable reimbursement models are urgently needed to enable broad accessibility and maximize the clinical utility of this strategy.

### 3.2 Activation and Inhibition of Key Signaling Pathways

Scarring has been identified as an inherent trait of a specific fibroblast subpopulation. The extent of fibrosis in regenerated tissue is governed by the dynamic interconversion and coordinated actions of these distinct fibroblast lineages [34]. In skin tissue, both En1 lineage‐positive fibroblasts (EPFs) and En1 lineage‐negative fibroblasts (ENFs) are present. While EPFs are primarily responsible for fibrotic tissue remodeling, ENFs represent the predominant fibroblast population in the dermis and do not contribute to scar formation. Notably, during both wound healing and developmental processes, ENFs can transition into EPFs under specific regulatory cues [35].

**FIGURE 5:**
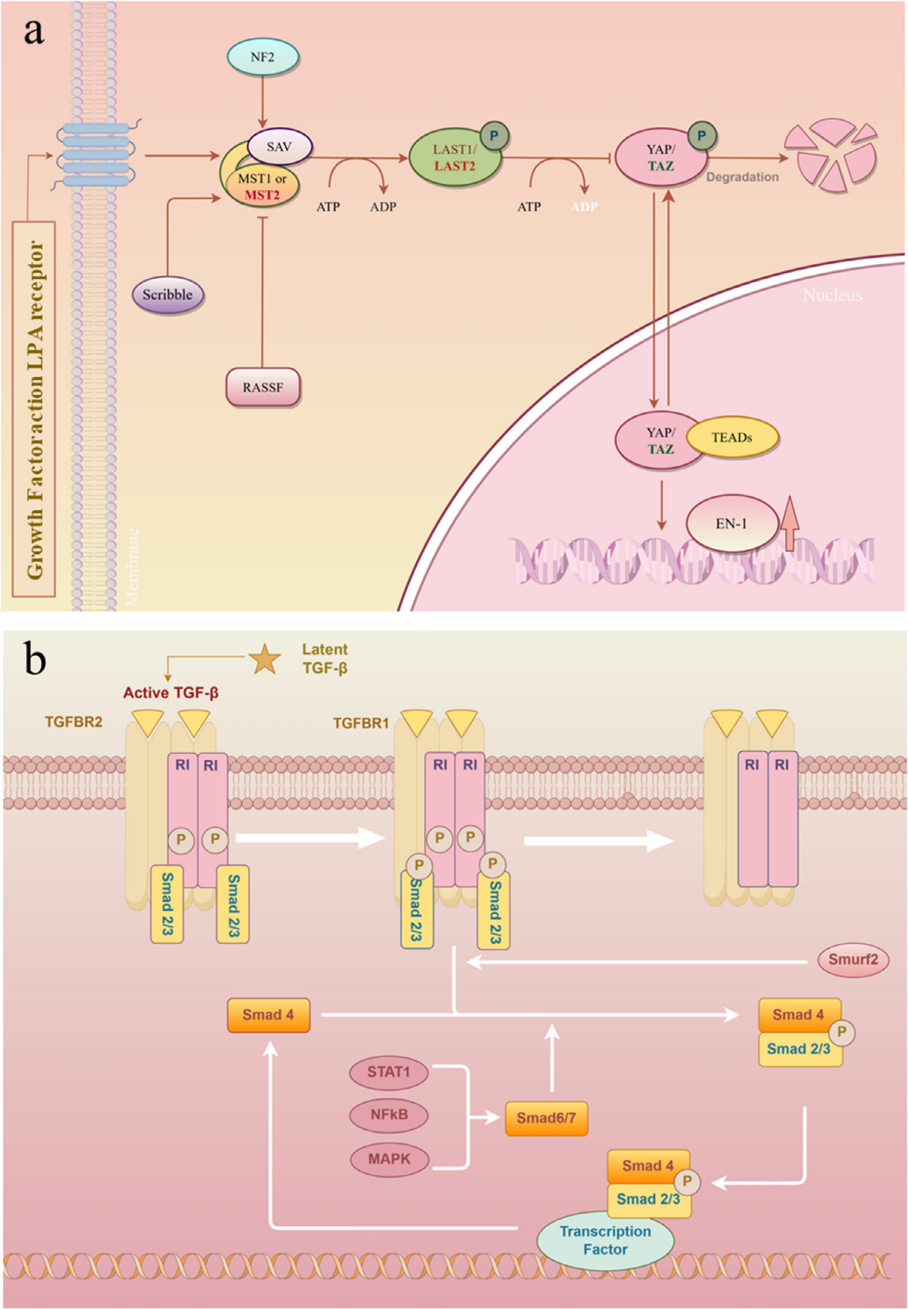
Key signaling pathways in scarless wound healing. (a) YAP signaling pathway. (b) TGF‐β/Smad signaling pathway.

Leveraging this cellular plasticity, Mascharak et al. explored the upstream regulatory mechanisms that drive ENF‐to‐EPF conversion. They found that in human skin, mechanical forces during wound healing activate the Engrailed‐1 signaling pathway in ENFs, thereby inducing their transformation into EPFs [36]. To therapeutically intervene in this process, the researchers targeted the mechanotransduction cascade by inhibiting the Yes‐associated protein (YAP) signaling pathway, a key effector of mechanical stress (Figure 5a). This blockade effectively suppressed Engrailed‐1 activation, reduced fibrotic conversion, and facilitated the regeneration of functional skin structures. These findings not only elucidate a pivotal regulatory axis in fibroblast fate determination but also highlight the therapeutic potential of modulating mechanosensitive pathways in promoting scar‐free tissue repair [37]. YAP is a key transcriptional coactivator and effector of the Hippo signaling pathway, playing a pivotal role in the regulation of cell proliferation, apoptosis, and lineage specification. As a mechanosensitive molecule, YAP activity is tightly modulated by extracellular mechanical cues, including matrix stiffness, cell density, and cytoskeletal tension. Under conditions of increased ECM rigidity or cell spreading, YAP translocates into the nucleus and drives the transcription of genes related to proliferation and matrix production. In the context of wound healing, YAP is dynamically activated in response to mechanical stress at the injury site. It regulates fibroblast activation, promotes myofibroblast differentiation, and modulates the synthesis of ECM proteins such as collagen. While transient activation of YAP is beneficial for early wound closure and tissue remodeling, sustained or excessive YAP activity is closely associated with pathological fibrosis and scar formation. Studies have shown that aberrant nuclear localization of YAP in fibroblasts leads to the upregulation of profibrotic genes, enhanced matrix deposition, and persistent fibrotic remodeling. Conversely, inhibition of YAP signaling has emerged as a promising strategy to mitigate fibrosis and promote regenerative healing. For instance, blocking YAP activity can suppress the conversion of non‐fibrotic fibroblast subtypes (e.g., ENFs) into fibrogenic phenotypes (e.g., EPFs), thereby reducing collagen overproduction and preventing scar tissue formation. Furthermore, YAP inhibition has been shown to facilitate the regeneration of skin appendages, such as hair follicles and glands, which are typically absent in fibrotic scar tissue. These findings underscore the dual role of YAP in balancing tissue regeneration and fibrosis, and highlight its therapeutic potential as a mechanotransduction target in scarless wound healing [38].

Mascharak also reported that fibroblasts upregulate stem cell and developmental pathways during regeneration. YAP‐inhibitory fibroblasts upregulate the transcriptional repressor GATA binding 1 (Trps1) and activate Wnt signaling, indicating that the Trsp1 and Wnt signaling pathways are beneficial for reducing skin fibrosis. Subsequent experiments revealed that Trsp1 is widely expressed in regenerative fibroblasts (Col1a1+ fibroblasts) and plays a role in promoting hair follicle regeneration and reducing ECM deposition in YAP‐inhibited wounds. Trsp1 plays a key role in the process of YAP‐mediated inhibition of scarless skin regeneration [39, 40].

Reducing the activation of fibroblasts by lowering mechanical stress and downregulating the expression of profibrotic cytokines is another effective strategy for inhibiting scar formation. Silk fibroin (SF) MNs physically intervene in wounds, and SF MNs can reduce the transmission of intracellular mechanical signals by weakening the integrin‐FAK signaling pathway and downregulating the expression of TGF‐β1, α‐smooth muscle membrane (α‐SMA), type I collagen, and fibronectin. This leads to a low‐stress microenvironment that helps reduce scar formation [41]. An imbalance in the TGF‐β/Smad signaling pathway is one of the key factors leading to scar formation and fibrosis (Figure 5b). This imbalance can cause abnormal collagen deposition, increasing the ratio of type I/III collagen and leading to the formation of abnormally cross‐linked collagen fiber bundles. TGF‐β plays an important role in inducing the myofibroblast phenotype, which leads to excessive collagen deposition in the wound and causes wound contraction [42]. Therefore, reducing the production of TGF‐β is a commonly used and effective antiscarring method.

**FIGURE 6:**
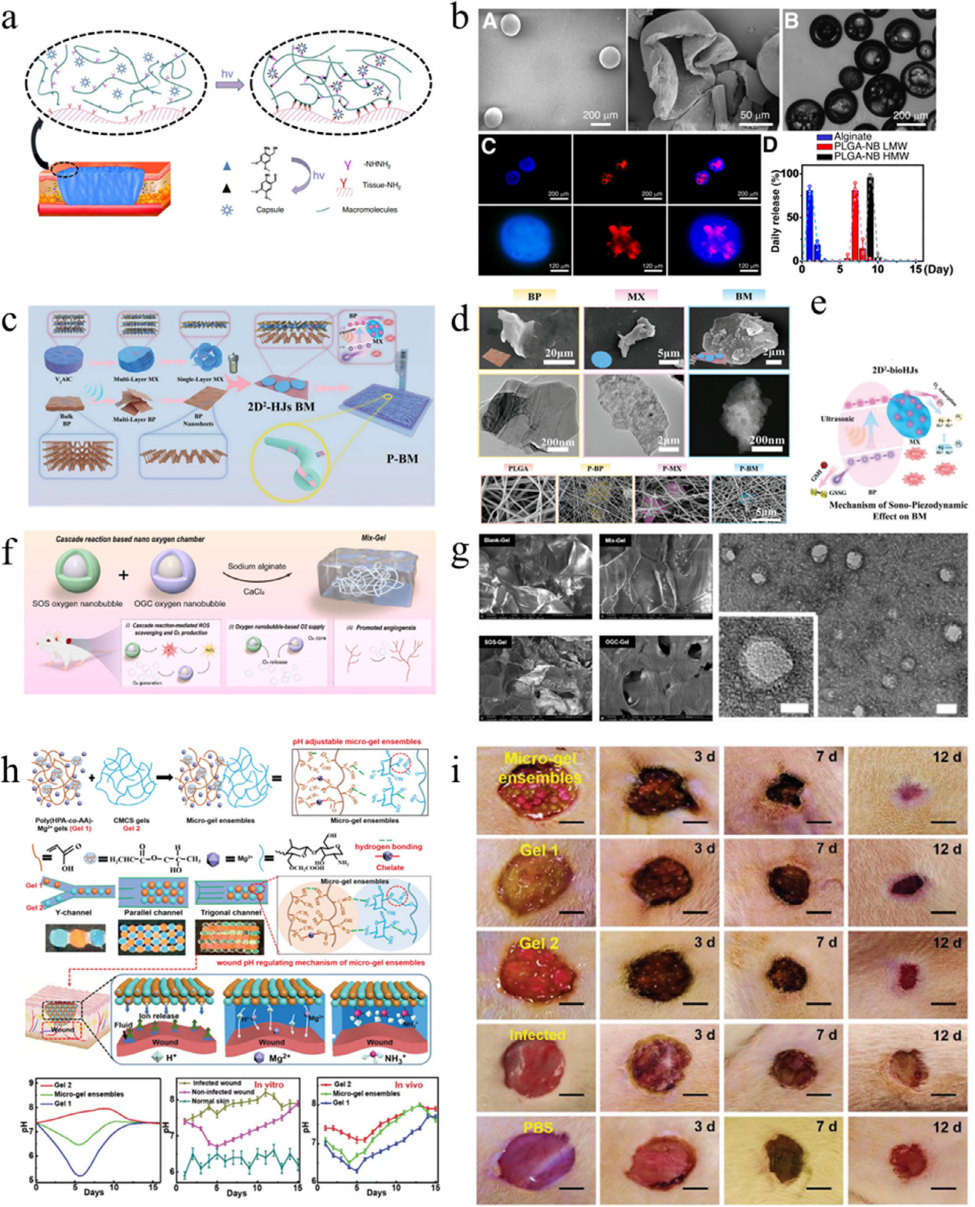
Strategies for improving the wound microenvironment to promote scarless wound healing. (a) Preparation of hydrogels containing PLGA‐NB capsules. (b) Drug release of PLGA‐NB capsules [43]. Copyright 2021, Springer Nature. (c) Schematic diagram of the intelligent nanocatalytic membrane. (d) FE‐SEM and TEM images of different nanosheets. (e) The working mechanism of 2D2‐bioHJS [56]. Copyright 2024, Wiley‐VCH. (f) Formation and application of Mix‐Gel and the mechanisms of enhanced healing. (g) Characteristics of the hydrogels [59]. Copyright 2024, Elsevier. (h) The structure of the microgel ensembles. (i) Photographic documentation of wound healing [61]. Copyright 2022, Wiley‐VCH.

A wound dressing based on an integrated photocrosslinking strategy and a microcapsule platform for the pulsed release of TGF‐β inhibitors can achieve precise control of the drug release time and location (Figure 6a,b) [43]. Previous studies have shown that inhibiting TGF‐β signaling during the early stages of wound healing can affect the epithelialization process and may have negative effects on chronic or difficult‐to‐heal wounds. Inhibition during the later stages of wound healing (6–13 days) can significantly improve scar outcomes [44]. To control the release time, PLGA‐NB capsules encapsulating TGF‐β inhibitors were used. Drug release experiments showed that the microcapsules released the drug after 6 days, which is within the effective time range for TGF‐β signal inhibition to improve scarring. Skin healing is a complex dynamic process, so designing a drug delivery system that can release different factors at different time points represents a significant advancement in scarless wound repair and can also be applied to other tissue regeneration fields.

The transformation of fibroblasts into myofibroblasts is a key step in scar formation and is regulated by epidermal growth factor receptor (EGFR) signaling in dermal fibroblasts (dFBs) [45]. The epidermal growth factor receptor EGFR and hyaluronic acid receptor CD44 jointly regulate the MAPK/ERK pathway, activating Ca2+/calmodulin‐dependent protein kinase II (CaMKII) and leading to the activation of myofibroblasts and collagen deposition [46]. Therefore, blocking the activation of the myofibroblast signaling pathway is crucial for reducing scar formation.

In skin repair, adipogenic progenitors form myofibroblasts rather than differentiate into adipocytes [47], which is detrimental to wound repair. Dermal adipocytes activate skin inflammation and improve skin repair efficiency. After skin injury, adipocytes at the wound site undergo lipolysis, releasing intracellular fatty acids into the wound environment [48]. Subsequently, due to lipolysis, adipocyte‐derived cells dedifferentiate, forming various myofibroblasts. Therefore, controlling the dedifferentiation or reversible dedifferentiation pathways of dermal adipocyte lineages can regulate the cellular composition of regenerative tissue. By employing single‐cell RNA sequencing on developing or injured mouse skin, dFBs were classified into distinct non‐adipogenic and adipogenic states [49]. Through biosignal analysis, WNT‐β‐catenin and IL‐1‐NF‐kB were found to be the top signaling pathways related to fat formation and negatively related to fat formation, respectively. The regeneration of adipocytes at the wound site is mediated by the IL‐1R‐NF‐kB‐CREB signaling axis. In contrast, the activation of the WNT signaling pathway inhibits the adipogenic potential of dFBs, promoting the lipolysis of mature adipocytes and dedifferentiation, thus promoting the formation of myofibroblasts. This study reveals the molecular mechanism underlying the plasticity of dermal adipocyte lineages and suggests that reducing the lipolysis of dermal adipocytes can reduce the generation of myofibroblasts at the wound site, thereby reducing collagen deposition, and proposes potential therapeutic targets for scarless wound healing.

Several pharmacological agents that target fibrotic signaling pathways have been applied clinically for scar treatment, primarily focusing on the inhibition of key fibrosis‐related pathways such as the TGF‐β/Smad pathway, the Wnt/β‐catenin pathway, the angiotensin II signaling pathway, and PDGF pathway, along with their downstream mechanisms involving fibroblast activation and collagen synthesis. Tazarotene, a retinoid commonly used in the treatment of psoriasis and acne, has been shown to activate the retinoic acid receptor (RAR)/retinoid X receptor (RXR) signaling axis, thereby indirectly suppressing TGF‐β signaling and attenuating collagen synthesis in scar tissue. It has been applied topically for hypertrophic scars and is supported by early clinical evidence indicating its potential to inhibit scar formation. Fresolimumab, a TGF‐β‐neutralizing antibody, inhibits the binding of TGF‐β to its receptors, whereas SB‐431542, a selective TGF‐β receptor I (TGF‐βRI) inhibitor, blocks Smad‐mediated signaling, thereby reducing the differentiation of fibroblasts into myofibroblasts. However, these agents have so far been evaluated primarily in clinical trials for systemic fibrotic diseases, such as systemic sclerosis, and have not yet been widely adopted for cutaneous scar treatment.

Most precision‐targeted anti‐fibrotic agents remain in the preclinical or early clinical stages of development. Currently, few agents demonstrate definitive anti‐scar efficacy and widespread clinical utility for skin fibrosis. This limitation largely stems from the pleiotropic roles of core fibrotic pathways such as TGF‐β, Wnt, and PDGF, which not only regulate scar formation but are also critically involved in physiological processes, including immune homeostasis, tumor surveillance, and angiogenesis. Systemic inhibition of these pathways may thus result in adverse outcomes such as immunosuppression, increased cancer susceptibility, or impaired tissue function. Notably, systemic administration of TGF‐β inhibitors has been associated with nephrotoxicity and cardiotoxicity, posing challenges to the implementation of individualized and precisely controlled antifibrotic therapy. Consequently, achieving high local efficacy with minimal systemic exposure remains a pivotal challenge in drug design. This necessitates the development of site‐specific and controllable delivery platforms, such as nanocarrier systems or stimuli‐responsive release materials, to mitigate off‐target effects caused by broad pathway inhibition and to enhance the therapeutic precision of antifibrotic interventions.

### 3.3 Regulation of Wound Microenvironment

The microenvironment of a skin wound can be divided into internal and external microenvironments based on spatial location [50]. The external microenvironment refers to the microenvironment outside the wound surface and mainly includes environmental temperature, humidity, pH, oxygen concentration, and pressure on the wound surface. The internal microenvironment mainly refers to the state of factors such as cells, cytokines, and the ECM within the wound. The external microenvironment indirectly affects the state of the internal microenvironment, and the internal microenvironment is the receptor for accelerating wound healing or reducing scar formation by improving the external microenvironment. The interaction and cooperation between the internal and external environments are crucial for wound healing [51].

The balance between the production and degradation of the ECM in the internal microenvironment regulates the wound healing process. Excessive production or degradation of the ECM can lead to hypertrophic scars and chronic ulcers, respectively. Targeting key factors implicated in ECM production and degradation, including TGF‐β, myofibroblasts, TIMPs, and MMPs, holds promise for enhancing wound healing and mitigating scar formation. The MMP protein family catalyzes the dissolution of key ECM components, including elastin, collagen, fibronectin, and laminin, and regulates ECM remodeling during wound healing [52]. The mitogen‐activated protein kinase (MAPK) pathway is a key signaling pathway regulating MMP protein expression and is a therapeutic target for wound healing disorders. Intravenous injection of exosomes from human adipose‐derived mesenchymal stem cells (ASC‐Exos) can activate the extracellular‐regulated protein kinase/MAPK (ERK/MAPK) pathway, increase the expression of matrix metalloproteinase‐3 (MMP3) in skin dFBs, increase the ratio of MMP3 to MMP1, and increase the ratio of type III collagen to type I collagen, thus reducing scar formation [53]. Another type of exosome from adipose‐derived stem cells, ADSC‐EXOs, can regulate the inflammatory response, promote skin epithelialization, and regulate collagen remodeling, which also has a good effect on reducing scarring [54]. Stem cell exosomes not only regulate cell differentiation but are also less likely to be rejected by the immune system and have homing effects, making them an advantageous strategy for wound healing treatment. In addition, some natural polymer materials have structures or functional properties similar to those of the ECM, so they can reconstruct the ECM. For example, chitosan, collagen, and hyaluronic acid (HA) all have good biocompatibility and are currently used as biomaterial scaffolds or delivery carriers for wound repair [55].

In the wound microenvironment, ROS derived from various sources play distinct roles across different phases of wound healing, exhibiting stage‐specific concentration levels and physiological functions. Proper ROS levels are crucial for facilitating wound repair, while excessive accumulation may lead to tissue damage and chronic inflammation. During the hemostasis phase, ROS levels remain relatively low and are primarily released by platelets, contributing to thrombus formation and signal transduction. In the inflammatory phase, ROS production markedly increases as neutrophils and macrophages generate large quantities of ROS to eliminate pathogens, initiate immune responses, and amplify inflammation. As the wound progresses to the proliferative phase, ROS levels gradually decline. Maintaining a low but controlled concentration of ROS during this phase supports the proliferation of keratinocytes and fibroblasts, as well as angiogenesis; however, excessive ROS can impair cellular functions. In the final remodeling phase, ROS levels approximate physiological baseline, where ROS participate in the regulation of MMPs, thereby facilitating collagen degradation and tissue remodeling. After skin injury, locally produced ROS can act as secondary messengers for immune cells and nonlymphoid cells. Low concentrations of ROS can clear bacteria and some invading microorganisms, but excessive ROS can aggravate inflammation, cause tissue oxidative damage, and exacerbate scar formation. The optimal level of ROS is necessary for fighting invading microorganisms and cell survival, so simply clearing ROS is not a reasonable method. The design of smart biomaterials capable of dynamically responding to ROS levels has become a key research focus for promoting wound regeneration and preventing pathological scar formation. Such intelligent materials should possess ROS‐responsive degradability; for example, by incorporating ROS‐sensitive chemical linkages (e.g., thioether, selenide, or boronic ester bonds) into the material matrix, the structure can undergo cleavage in high‐ROS environments, thereby enabling controlled degradation and on‐demand drug release. This property is particularly valuable during the inflammatory phase, where it facilitates the targeted delivery of anti‐inflammatory or antioxidant therapeutics. More importantly, these materials should also exhibit intrinsic ROS‐scavenging capabilities. This can be achieved by covalently conjugating or embedding ROS‐neutralizing agents such as glutathione, superoxide dismutase (SOD), phenolic antioxidants, or metal‐organic framework (MOF)‐based nanozymes to mitigate oxidative stress and restore redox homeostasis. In addition, multilayered or sequential drug delivery systems can be engineered to achieve stage‐specific therapeutic effects, initially releasing antioxidants to control inflammation, followed by the release of pro‐regenerative factors, such as vascular endothelial growth factor (VEGF) and basic fibroblast growth factor (bFGF) to support fibroblast migration and angiogenesis. Such programmable delivery strategies enable precise temporal regulation of the wound healing process. A smart nanocatalytic film composed of poly(lactic‐*co*‐glycolic acid) (PLGA) and black phosphorus/V2C MXene heterojunctions generates a large amount of ROS through ultrasound, clearing drug‐resistant bacteria, and clearing ROS produced by sono‐piezodynamic therapy (SPT) after ultrasound stops, achieving both antibacterial and anti‐inflammatory effects (Figure 6c–e) [56]. This programmed control of ROS generation and elimination demonstrates the potential of this nanocatalytic film in addressing the challenge of treating persistent bacterial invasion wounds, greatly increasing the potential and application of antimicrobial materials and SPT in wound repair and providing guidance for programmed control strategies [56].

There are many factors affecting the external microenvironment, which also provides more possibilities for treatment. Skin barrier damage can cause increased water vapor evaporation and tissue fluid loss at the wound site. Using dressings or wound chambers to maintain the humidity of the external wound microenvironment can accelerate wound healing and reduce the scar area [57]. Oxygen is essential for cell proliferation, angiogenesis, and protein synthesis. Hypoxia can hinder tissue regeneration, delay wound healing, and increase the risk of infection by hindering cell proliferation, angiogenesis, and re‐epithelialization. Biomaterials currently used for oxygen delivery therapy include catalase, nanoenzymes, peroxidases, peroxidized proteins, peroxidized calcium, and perfluorocarbon materials [58]. A hydrogel dressing based on a cascade enzyme reaction with a nanocarrier encapsulates excess superoxide in the wound environment through SOD into hydrogen peroxide, which is then converted into oxygen by catalase (Figure 6f,g). This enzyme cascade effectively reduces inflammation at the wound site and alleviates hypoxia symptoms [59].

pH is one of the basic parameters of the wound microenvironment and affects cell proliferation, functional expression, and the healing cycle, including inflammation and collagen production, throughout the entire wound healing process. Intact skin is naturally acidic, with a pH of 4–6. When injured, the pH at the wound surface increases due to microvascular leakage and is close to the physiological pH suitable for bacterial infection (7.4) [60]. Microfluidic technology‐based microsphere hydrogels precisely regulate pH to improve wound healing. The microgel system is composed of carboxymethyl chitosan (CMCS) gel and poly(hydroxypropyl acrylate‐*co*‐acrylic acid)‐magnesium ion (poly(HPA‐*co*‐AA)‐Mg^2+^) gel, which can release and absorb hydrogen ions (H^+^) at different stages of healing to respond to the evolution of the wound microenvironment (Figure 6h,i) [61]. In the early stage of the wound, hydrogen ions are released to create an acidic environment to inhibit bacterial infection and promote angiogenesis. In the later stage, hydrogen ions are absorbed to create an alkaline environment conducive to cell proliferation and skin reshaping. This research can continuously and intelligently regulate the pH of the wound microenvironment at four stages of healing, making the wound microenvironment controllable and maintaining it within a range conducive to healing. This not only provides inspiration for how biomaterials can more accurately regulate wound acidity but also provides strategies for continuously and dynamically and intelligently controlling the wound microenvironment.

Compared to therapeutic strategies centered on immune modulation or inhibition of fibrotic signaling pathways, approaches that improve the wound microenvironment to reduce scar formation are more widely applied in clinical practice and generally exhibit higher safety and patient compliance. Among these, silicone‐based dressings are among the most frequently utilized interventions. These dressings form a semi‐permeable protective film over the wound surface, which effectively maintains a moist healing environment, reduces transepidermal water loss (TEWL), and significantly lowers local tissue tension and mechanical stress. Consequently, the activation of fibroblasts and excessive collagen deposition are suppressed, thereby improving scar appearance. Other interventions that operate via similar mechanisms include hyaluronic acid‐based hydrogels and other moisture‐retentive medical dressings, which not only preserve wound hydration and promote keratinocyte migration and re‐epithelialization but also provide a physical barrier that minimizes external irritation during healing. In addition, some hydrogel platforms can serve as drug delivery vehicles, enabling targeted and controlled release of therapeutic agents at the wound site. Pressure therapy, another classic microenvironment‐modulating technique, is commonly combined with silicone gel sheets and is widely employed in the prevention and management of hypertrophic scars following extensive burns. This therapy applies sustained external compression to the wound, reducing capillary perfusion and local oxygen tension, which indirectly inhibits fibroblast‐mediated collagen synthesis and slows aberrant scar tissue proliferation. Furthermore, a class of topical antioxidants capable of scavenging excess ROS and modulating oxidative stress at the wound site has also gained traction in adjunctive clinical treatments. Agents such as glutathione (GSH), SOD, vitamin C, and vitamin E have demonstrated efficacy in attenuating ROS‐induced fibroblast activation and pathological collagen accumulation. These formulations are particularly suited for intervention during the inflammatory phase of wound healing, where oxidative stress is often elevated.

However, it should be noted that these microenvironment‐targeted therapies primarily act on the external physical and biochemical environment of the wound. Their core mechanisms rely on suppressing exogenous stimuli, maintaining a moisture barrier, and modulating local mechanical tension to improve scar morphology. While these approaches show favorable outcomes in reducing scar hypertrophy and alleviating related symptoms, they are generally insufficient to achieve functional skin regeneration, such as the reconstitution of skin appendages, including hair follicles and sweat glands. Therefore, in future therapeutic paradigms, these microenvironment‐modifying interventions are best suited as adjunctive treatments, complementing regenerative strategies such as stem cell therapy, tissue engineering, and signaling pathway inhibitors [62]. This integrative approach holds promise for achieving the dual goals of both structural remodeling and functional restoration in scar treatment.

### 3.4 Regeneration of Skin Appendages

Skin appendages include sebaceous glands, hair, nails, and sweat glands, all of which are derived from the ectoderm. After skin injury and repair, skin appendages may lose some or all of their functions, and their numbers are reduced, making the regenerated skin area unable to grow hair, sweat, and so on. Therefore, the regeneration of skin appendages is key to the regeneration of skin structure and function. In addition, appendages not only help with skin function regeneration but also help regenerate normal skin tissue structures. Studies have shown that new hair follicles around the wound site are surrounded by a layer of fat, and the surrounding skin tissue approaches normalization. Subsequent studies have shown that new hair follicles secrete a growth factor called bone morphogenetic protein (BMP), which can convert fibroblasts into adipocytes to improve scarring [63]. Under normal circumstances, HFSCs do not participate in the self‐renewal of the epidermal layer, but when the skin is injured, HFSCs can be activated and migrate to the wound edge to help repair the wound and regenerate the skin. Because of their multidirectional differentiation potential, they can aid in blood vessel regeneration and sebaceous gland regeneration at the wound site [64]. Therefore, promoting the regeneration of skin appendages can help restore the structure and function of the skin and reduce the generation of scar tissue by normalizing new skin tissue.

There are two main methods of hair follicle regeneration: hair follicle cycling regeneration and hair follicle neogenesis [65]. Hair follicles cycle back and forth between the anagen and telogen phases. By activating HFSCs, they can enter the anagen phase from the telogen phase, achieving hair follicle cycling regeneration. A key factor, a defect in FoxC1 in HFSCs, leads to a shorter resting time, resulting in a significantly shortened rest time between hair cycles during enhanced hair cycling, which accelerates the consumption of HFSCs and affects hair regeneration in aging mice [66]. Wnt signaling plays a crucial role in hair follicle development, and its activation helps maintain the hair follicle growth cycle and regulates the proliferation and differentiation of HFSCs. Damaged skin neogenesis of follicular parts originates from HFSCs; that is, HFSCs also have the potential to form epithelial cells [67]. Inhibiting the Wnt signaling pathway before wound epithelialization does not result in the formation of new follicles, but when Wnt ligands are overexpressed in the epidermis, the number of regenerated hair follicles increases, indicating that regulating the Wnt pathway can rebuild damaged hair follicles and achieve scarless healing. Hair follicle neogenesis refers to the generation of new hair follicles by other sources of stem cells that differentiate into cell lineages with hair follicle phenotypes. A key gene encoding a transcription factor, SOX9, is the main regulator of the conversion of embryonic epidermal stem cells (Epi‐SCs) into HFSCs. SOX9 affects the differentiation of HFSCs into different cell lineages by regulating the expression of other genes, thus maintaining the normal growth and regeneration of hair follicles [68]. Implanting epidermal stem cells (Epi‐SCs) and skin‐derived precursor cells (SKPs) into the wound can rebuild hair follicles and sebaceous glands [69], and by analyzing the signal communication between these two types of cells, it has been found that the PI3K‐Akt signaling pathway is significantly upregulated in Epi‐SCs, and several growth factors and cytokines that may activate PI3K are expressed at higher levels in SKPs. When the PI3K‐Akt pathway is inhibited, hair follicles no longer form, indicating that the PI3K/Akt pathway is a potential signaling pathway for hair follicle regeneration [70].

Recent research has increasingly revealed the important impact of the local immune microenvironment on hair growth, suggesting that immune cells are new therapeutic targets. Regulatory T cells (Tregs; CD4+ Foxp3+) play important roles in both wound repair and hair follicle reconstruction [71]. Tregs accumulate around HFs, activate HF stem cells, and promote the transition of HFs from a resting (telogen) state to a regenerative (anagen) state. Therefore, locally recruiting Tregs to activate HFs is a possible therapeutic strategy. Inspired by the physiological feedback in skin immunity that inhibits inflammation triggered by microorganisms, a “revealing” oligosaccharide biomaterial with specific activity has been developed to recruit Tregs around HFs, thereby accelerating hair growth in mice [72]. One of the oligosaccharides obtained by the enzymatic cleavage of glucomannan polysaccharide, hexose (OG6), can induce macrophages to express the chemokine CCL5, which recruits Treg cells to the wound site. Although it is known that Treg cells can regulate HF cycles and hair regeneration, due to the complexity of immune tissue crosstalk and the difficulty in precisely controlling Treg activity, there have been few attempts to manipulate Treg activity for treatment; moreover, direct injection of exogenous CCL5 can cause inflammation, and wounds may undergo premature degeneration [73]. This study used an oligosaccharide material to drive the endogenous release of the chemokine CCL5, achieving the controlled release of chemokines without side effects; thus, this material represents an unconventional, biomaterial‐based, precise immunotherapy method for hair loss and provides more possibilities for the application of polysaccharide materials.

The regenerative potential of sweat glands (SGs) decreases after severe burns or trauma. Sweating is the main function of sweat glands, and the realization of this function also requires precise cooperation between the surrounding microvasculature and the nervous system. Therefore, restoring SG function requires not only the integrity of the gland itself but also the reconstruction of the surrounding vascular tissue, which still presents many challenges in clinical treatment. Collagen triple helix repeat containing‐1 (CTHRC1) was first discovered in vascular repair. Experiments have shown that the knockout of Cthrc1 reduces the expression and function of vascular development genes, and the absence of Cthrc1 leads to disorders and reductions in the microvascular network around SGs. Therefore, CTHRC1 can significantly enhance SG function by inducing reconstruction of the adjacent vascular network [74]. This study provides a new therapeutic target for the regeneration of SG function. Sweat gland cells are difficult to isolate from skin tissue, so it is challenging to achieve large‐scale culture in vitro. Currently, gene reprogramming can induce sweat gland induction, so discovering key transcription factors that induce cell differentiation is important [75]. Irf6 is a key regulatory factor for the proliferation and differentiation of keratinocytes and is essential for epidermal development. In a mouse burn model, Irf6 was shown to guide the lineage differentiation of epidermal progenitor cells and promote limited sweat gland regeneration. Transplanting induced SG cells to the burn site restored the sweating function of damaged skin and promoted reconstruction of the surrounding microvasculature [76]. Overexpression of the transcription factor FoxC1 can reprogram epidermal cells to induce the formation of functional sweat gland‐like cells (iSGCs). Transplanting iSGCs into a mouse burn model was found to be beneficial for wound repair and sweat gland regeneration, restoring the sweat gland function of damaged skin [77]. The combination of somatic cell reprogramming through the overexpression of various transcription factors (TFs) has been established as a new means to induce various somatic cell types, especially somatic cells, which are not easy to culture in vitro. This approach is also an effective therapeutic means for the reconstruction of skin appendages such as sweat glands and hair follicles.

In current clinical practice, strategies aimed at regenerating skin appendages to restore both the structure and function of scarred tissue remain in the early exploratory stage. At present, the most clinically feasible approach is follicular unit transplantation, which is primarily employed in cases of cicatricial alopecia or scar‐induced baldness. This technique involves transplanting follicular units from healthy donor sites into scarred areas, thereby facilitating local follicle reconstruction and regeneration. Clinical observations have demonstrated its potential to restore localized hair growth and improve the aesthetic and textural qualities of scar tissue. However, its application is largely limited to small, morphologically stable scars, as extensive scarring typically exhibits significant ECM stiffening, aberrant immune cell infiltration, and persistent fibrotic activity, all of which contribute to a regeneration‐inhibitory microenvironment that severely impairs the regenerative capacity of hair follicles and other skin appendages. Against this backdrop, stem cell and exosome‐based therapies have emerged as promising strategies in regenerative medicine and are under active investigation to overcome the limitations of conventional treatments. These approaches capitalize on the multipotent differentiation potential of stem cells and their paracrine effects, which include the secretion of various growth and signaling factors such as VEGF, FGF, and insulin‐like growth factor that modulate the local microenvironment and promote the regeneration of appendages such as hair follicles and sweat glands. Commonly employed stem cell types include mesenchymal stem cells (MSCs), epidermal stem cells, and dermal papilla cells. Simultaneously, exosomes derived from these stem cells, as representative cell‐free therapeutic agents, are gaining traction due to their high bioactivity, low immunogenicity, and ease of storage. They are progressively being formulated into topical delivery systems, such as sprays, gels, and microneedle patches, thus enhancing their clinical practicality and application flexibility. Despite the encouraging outcomes observed in preclinical animal models, the clinical translation of these therapies still faces considerable hurdles. Chief among these is the challenge of precise delivery of bioactive agents to specific microenvironmental niches, such as the follicular stroma or basement membrane zones. Scar tissue is often characterized by low vascularity, limited microchannel availability, and ECM densification, all of which impede the penetration and targeted localization of therapeutic agents. Furthermore, many bioactive molecules exhibit short in vivo half‐lives and are prone to enzymatic degradation or off‐target diffusion, resulting in insufficient or unstable therapeutic effects. Therefore, there is an urgent need to develop intelligent delivery systems or functional scaffolds that possess biological responsiveness, targeting capabilities, and controlled release properties, thereby ensuring the stability and sustained bioactivity of therapeutic components at the target site.

In conclusion, although strategies for skin appendage regeneration hold significant promise, their clinical implementation requires overcoming multiple barriers along the “bench‐to‐bedside” translation pathway. Future efforts should focus on the development of precision delivery platforms, comprehensive safety evaluation of stem cell‐based products, intelligent design of bioactive materials, and the establishment of standardized clinical endpoints. Such multidisciplinary advancements are essential for the transition of these promising approaches from experimental validation to routine clinical application.

### 3.5 Cellular Reprogramming

Reprogramming refers to the alteration of cell fate via epigenetic modifications without affecting the underlying gene sequence. The famous “Yamanaka factors,” namely, Oct3/4, Sox2, c‐Myc, and Klf4, can convert mature somatic cells into pluripotent stem cells, allowing cells to rejuvenate. Scientist Shinya Yamanaka introduced these four factors into mouse skin cells and ultimately obtained pluripotent stem cells with strong self‐renewal and differentiation potential, which can develop into various tissue cells. These cells were named induced pluripotent stem cells (iPSCs). With increasing in‐depth research on cellular reprogramming, reprogramming wound‐resident skin cells into corresponding functional cells has become a therapeutic strategy. During the wound repair process, keratinocytes migrate from the adjacent epidermis to the site of injury, resulting in wound re‐epithelialization [17]. Using reprogramming technology, mesenchymal cells (dFBs [hDFs] and adipose‐derived stromal cells [hADSCs]) around the wound can be reprogrammed into epidermal progenitor cells, forming new epithelial cells to heal the wound more quickly [78]. Although animal experiments have proven that this in situ reprogramming technology can achieve re‐epithelialization in the wound area, its application in clinical practice still faces many challenges, as the degree of inflammation at the wound site may affect the efficiency or outcome of reprogramming, and finding more effective gene delivery methods to enhance targeting, reduce off‐target risks, and improve safety is crucial. In addition, this research also proves the feasibility of single‐cell and three‐dimensional functional tissue regeneration in vivo, which can not only be applied to skin repair but also provide guidance for the repair of other organ tissues.

In cellular reprogramming, finding suitable donor cells and target cells is crucial for improving the efficiency and outcome of reprogramming. Suitable donor cells proliferate easily, providing enough programmable cells, and have good differentiation potential, as well as fewer gene mutations and fewer epigenetic modifications, to avoid gene mutations during reprogramming and ensure the effective expression of target cell functions [79]. Currently, commonly used donor cells include adipose tissue stem cells (ATSCs), fibroblasts, neural glial cells, and peripheral blood mononuclear cells [80, 81, 82, 83]. Keratinocytes (KCs) are abundant in the skin and have the characteristics of both donor cells and target cells. KCs can be reprogrammed into iPSCs by forced expression of cell factors (Oct4, Klf4, c‐Myc, and Sox2) [84]. In addition, in the wound microenvironment, differentiated epidermal cells can dedifferentiate into epidermal stem cells [85]. Therefore, reprogramming keratinocytes into stem cells with undifferentiated characteristics and then inducing stem cells to differentiate into target functional cells is a feasible strategy for achieving scarless wound repair using in situ reprogramming technology, with the advantages of easy operation and high safety. When keratinocytes are target cells, they can be derived from embryonic stem cells (ESCs), iPSCs, and fibroblasts. However, ESCs have ethical restrictions, so iPSCs can be used as an alternative, as they also have good differentiation potential and can be derived from various donor cells, greatly expanding the application scope of stem cells in regenerative medicine. Fibroblasts reprogrammed into KCs first need to be reprogrammed into iPSCs and then cultured with retinoic acid (RA) and BMP4 to allow them to differentiate into KCs [86]. The above findings indicate that reprogramming wound‐resident cells into functionally ideal cell types via cellular reprogramming technologies holds considerable promise. Current reprogramming strategies—including transcription factor induction, chemical stimulation, and small‐molecule compound intervention—have demonstrated potential applications in tissue engineering and regenerative medicine. Despite this progress, the clinical translation of reprogramming technologies remains challenging, as unresolved safety concerns such as genomic instability and tumorigenic risk continue to demand rigorous investigation.

**FIGURE 7:**
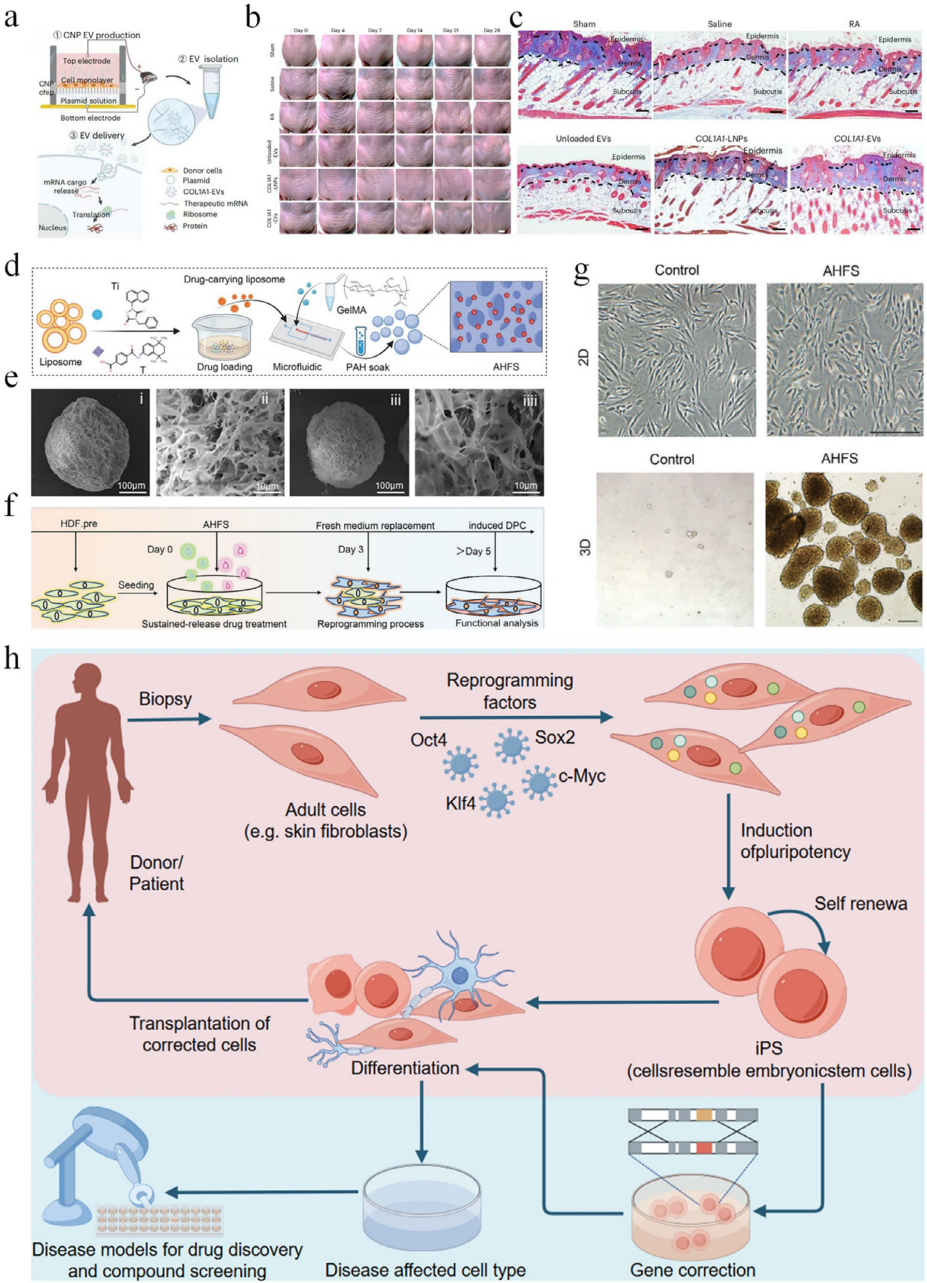
Strategies for promoting scarless wound healing through cellular reprogramming. (a) The fabrication of EVs. (b) The recording of wrinkle formation. (c) Mason staining image of slices [87]. Copyright 2023, Springer Nature. (d) The fabrication of AHFS microspheres. (e) SEM images of GelMA microspheres (i, ii) and AHFS microspheres (iii, iiii). (f) The AHFS microsphere‐based reprogramming procedure. (g) Images of the contrast of HDF and AHFS microsphere‐induced DPC. [89] Copyright 2023, Wiley‐VCH. (h) Schematic diagram of cell reprogramming.

Among these strategies, transcription factor‐based reprogramming—often mediated by the delivery of messenger RNA (mRNA)—has attracted increasing attention. In this context, the efficacy and safety of mRNA delivery systems are critical to therapeutic success. Lipid nanoparticles (LNPs) are the most widely used vectors; however, their application is often limited by issues such as suboptimal biodistribution, cytotoxicity, immunogenicity, and poor targeting specificity. To address these limitations, recent studies have explored alternative delivery systems. For example, cell‐derived extracellular vesicles (EVs) generated through cell nanopore technology have been employed to encapsulate mRNA encoding ECM component α1 type I collagen (COL1A1), thereby enhancing both delivery precision and regenerative efficacy (Figure 7a–c) [87]. The vesicle is delivered to the dermis through microneedles, which can reduce the production of wrinkles in aging mice and achieve long‐term endogenous collagen replacement, maintaining the strength, tightness, and elasticity of the skin over time. This research provides a new model for treating skin diseases or anti‐skin aging and has better biosafety and immunogenicity than LNPs. Compared to that of transcription factors, chemical induction reprogramming is more convenient and highly controllable. Chemical reprogramming methods synergistically target cell signaling pathways and epigenetic modifications of cell signaling pathways without the need for gene manipulation, greatly increasing clinical safety [88]. Shuaifei Ji and others designed a hydrogel microsphere that can reprogram cells, inducing dFBs at the wound site to undergo reprogramming into dermal papillary cells (DPCs) and reducing the transformation of fibroblasts into myofibroblasts to reduce scar formation and promote the regeneration of in situ hair follicles (Figure 7d–g) [89]. Moreover, by modifying the surface of the microspheres to have a positive charge, the microspheres have good adhesion at the wound site, avoiding the shortcomings of early invasive treatment. This research describes a typical process from screening chemical inducers to designing biomaterials for wound repair, opening up a new field for scarless treatment using chemical induction reprogramming and biomaterials. Cellular reprogramming technology has great application prospects in the field of tissue regeneration medicine. It is important to avoid the risks that reprogramming brings to human health while ensuring the quality of regeneration. A safe, precise, and highly controllable reprogramming method is necessary.

Cellular reprogramming offers a novel therapeutic perspective in regenerative medicine for scar reduction, particularly by enabling the restoration of dermal‐appendage structures and functional regeneration rather than merely improving cosmetic appearance. Currently, most strategies under investigation or in experimental phases for scar attenuation via cellular reprogramming focus on three main approaches: in situ transdifferentiation of fibroblasts, ex vivo reprogramming followed by cell transplantation, and chemical small‐molecule–based reprogramming (Figure 7h). The overarching goal of these methods is to reverse the fibrotic phenotype of scar tissue and promote the regeneration of functional skin components, including hair follicles and sweat glands. In situ cellular reprogramming involves the direct injection of transcription factors such as p63 and Klf4 into the scar tissue, allowing fibroblasts or myofibroblasts to be converted into other functional cell types, such as epidermal cells, follicular cells, or mesenchymal stem‐like cells, without the need for prior extraction or in vitro culture. This localized strategy is minimally invasive and allows precise targeting. However, many reprogramming protocols currently rely on viral vectors such as lentiviruses or retroviruses to deliver key transcription factors (e.g., OSKM: Oct4, Sox2, Klf4, c‐Myc), which pose significant biosafety concerns, including risks of genomic instability, insertional mutagenesis, and oncogenic transformation. Ex vivo reprogramming coupled with cell transplantation entails inducing patient‐derived scar fibroblasts into iPSCs or functional cell lineages in vitro, followed by selection, expansion, and reinfusion into the scar site. In parallel, chemical reprogramming using defined small‐molecule cocktails such as CHIR99021, valproic acid (VPA), RepSox, and forskolin has emerged as a virus‐free and gene‐editing–independent approach with higher safety margins and greater clinical translatability.

Nonetheless, even with chemical or non‐viral methods, challenges persist due to potential off‐target effects and unpredictable cell fate trajectories, which may result in aberrant structural outcomes or functional deficiencies. Moreover, the clinical translation of cellular reprogramming strategies remains hindered by the absence of ideal delivery platforms for localized factor administration. Most current approaches rely on direct injection or topical application, which lack sustained release capability and cellular specificity. The delivery of mRNA or protein‐based reprogramming agents further faces additional obstacles, including rapid degradation, strong immune recognition, and poor nuclear entry. Consequently, maintaining effective local concentration gradients and prolonged biological activity at the target site remains a major barrier in clinical implementation.

### 3.6 Cell/Tissue Engineering

Cellular skin substitutes have been engineered for diverse applications, ranging from wound healing to skin regeneration. Many of these models utilize primary cells as their foundational source. With a deeper understanding of human skin phenotypes and physiology, in vitro cell culture has progressed from the initial 2D to the current 3D cell culture. 3D cell culture not only provides a more powerful adhesion and proliferation space for cells but also allows cells to self‐assemble in vivo, simulating the multilayered tissue of the human body and achieving complete simulation of composition and structure [90, 91]. More realistic skin substitutes transplanted into the wound site have better repair effects. Currently, autologous stem cell transplantation is used to induce skin regeneration because it not only has good immunogenicity but also induces the aggregation and proliferation of in situ cells through the homing effect of stem cells, maximizing complete regeneration [92]. A human skin organoid has been designed that can achieve self‐assembly processes similar to skin development in vitro and has been successfully used for damaged tissue reconstruction. This is highly important for the development of cellular skin substitutes and complete wound repair [90].

**FIGURE 8:**
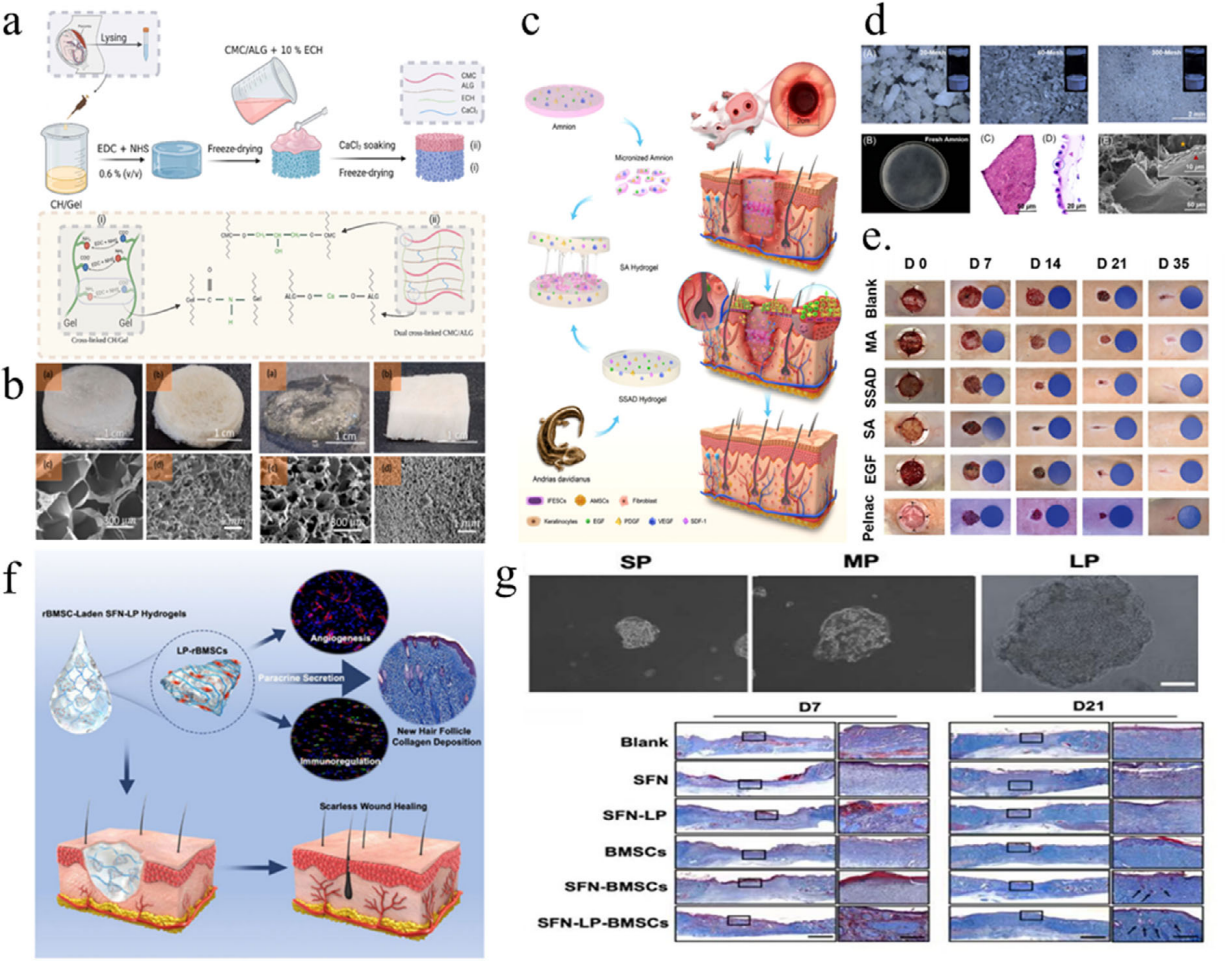
Strategies for scarless wound healing via cell/tissue engineering techniques. (a) The process of producing the bilayer hydrogel. (b) Images and SEM images of dermis and epidermis‐simulating scaffolds [93]. Copyright 2023, Elsevier. (c) The fabrication of SA. (d) Characterization of the materials. (e) Photographs of the wounds were recorded [94]. Copyright 2023, Wiley‐VCH. (f) The fabrication of the hydrogel. (g) Images of BMSC‐laden SP, MP, and LP particles after incubation for 24 h and tissue sections stained with Masson's trichome [96]. Copyright 2020, Wiley‐VCH.

Combining cells with biomaterials for tissue regeneration is the core of tissue engineering. Cells adhere to biomaterials (scaffolds) to create cell‐material complexes. As biomaterials degrade and are absorbed by the body, implanted cells persist in proliferating and secreting ECM, ultimately facilitating the formation of the desired tissue or organ. This process serves to repair trauma and restore function. For example, a multifunctional bilayer hydrogel containing human placental extract (HPE) simulates the structure of the epidermis and dermis of the skin. The upper layer of the hydrogel is a dressing layer with antimicrobial and cell proliferation support functions, and the lower layer is a cell matrix‐like layer that provides a good biological environment for cell growth. By continuously releasing HPE, inhibiting bacterial invasion, and promoting cell proliferation, the hydrogel accelerated the healing of full‐thickness wounds (Figure 8a,b) [93]. Another lizard mucus gel containing a micropowdered amniotic membrane was used to achieve efficient scarless skin regeneration (Figure 8c–e) [94]. Experiments have shown that after the gel is implanted, more skin appendages, such as sweat glands and hair follicles, are reconstructed, and collagen deposition in the dermis layer becomes more uniform, resulting in smaller scars. Both of these biomaterials are loaded with embryonic‐related biological components, which may be inspired by previous studies on scarless wound healing in fetuses [95]. However, compared to extracting components surrounding the fetus, exploring the biological mechanisms by which fetuses do not form scars is more applicable. Similarly, MSCs with differentiation potential are also used to induce skin regeneration. MSCs are loaded into hydrogels and dispersed into SNFs to form injectable gels, which are used for scarless hair follicle wound healing. MSCs have good self‐renewal ability and secrete active cytokines to promote angiogenesis and skin structure reconstruction (Figure 8f,g). [96] The use of autologous stem cells for transplantation and tissue regeneration has limited potential for immune rejection. Studies have shown that autologous adipose stem cell transplantation to the wound site significantly promotes skin healing and regeneration [97]. There are still many possibilities for the application of easily accessible autologous stem cells in tissue regeneration. Whether to transplant cells or combine cells with biomaterials to treat wounds, reduce immune rejection possibilities, and maximize skin structure restoration are necessary and will be important criteria for evaluating the therapeutic effects of tissue engineering in skin repair in the future.

The clinical translation of 3D cell culture and tissue engineering technologies for enhancing wound healing, tissue regeneration, and scar reduction still faces several substantial obstacles. These include low cell expansion efficiency, tissue heterogeneity, high manufacturing costs and long production timelines, a lack of long‐term clinical data, and variability in patient‐specific therapeutic responses. Therefore, it is imperative to establish strategic approaches to facilitate the advancement of these technologies into clinical practice. Scalable 3D culture system design is essential for improving cell proliferation rates. The incorporation of bioreactors enables large‐scale cell expansion while maintaining a dynamic culture environment, such as controlled shear stress and oxygen concentration that more closely simulates the in vivo milieu. Additionally, the use of microcarriers significantly increases the surface area for cell attachment, enhancing suspension culture efficiency, particularly for epithelial cells such as keratinocytes or stem cells. When designing biomaterials to replicate the structure of native skin, tissue heterogeneity presents a considerable challenge. This necessitates the co‐culture of multiple cell types, including keratinocytes, fibroblasts, endothelial cells, and melanocytes. Optimizing the co‐culture conditions to maintain the functionality and spatial organization of each cell population is critical for achieving a physiologically relevant construct. Challenges also persist in the downstream processes of quality control and standardized production of engineered skin substitutes. For transplantable products, the origin of the cells must be well‐defined to minimize immunogenicity, preferably using autologous or iPSC sources, and all materials must be rigorously screened for microbial contamination and pathogenic agents to ensure biosafety. Moreover, stem cell‐derived products require thorough evaluation of differentiation consistency and tumorigenic potential [98]. To improve patient accessibility and reduce costs, the development of ready‐to‐use or lyophilized storage forms is advantageous. Such formats enhance the stability of the product during transport and facilitate clinical application. Employing temperature‐controlled transportation systems and long‐term preservation solutions can further improve the availability and usability of engineered skin products, ultimately accelerating their clinical translation.

## 4 Potential Therapies

Many factors affecting scar formation, including the immune environment, key signaling pathways, and the wound microenvironment, have been identified. Corresponding strategies have been developed to improve each factor, but an increasing number of studies have shown that combined treatments considering multiple factors can achieve better results. This combined therapy will also be the starting point for designing treatment strategies in the field of regenerative medicine in the future.

### 4.1 Engineering Biomaterials for In Situ Tissue Regeneration

The primary objective of regenerative medicine is harnessing the intrinsic regenerative capabilities of mammals to reconstruct intricate tissue structures. The most recent advancement involves integrating the body's regenerative potential with biomaterials, a concept referred to as in situ tissue regeneration [99]. Engineered biomaterials can be used for immune modulation; as delivery carriers for cells, cytokines, proteins, etc. and as 3D biological scaffolds for cell culture in vitro or in vivo. By designing the structure and biochemical properties of biomaterials, specific immune cell types can be recruited, as mentioned earlier, using fMLP to specifically recruit neutrophils, inducing temporary high inflammation to promote rapid healing of chronic wounds. Hydrophilic scaffolds promote the adsorption of albumin, causing local macrophages to produce anti‐inflammatory cytokines, while hydrophobic materials are more conducive to the adsorption of immunoglobulins and stimulate macrophages to polarize into the M2 type [100]. Biomaterials with positive charges have been found to activate proinflammatory signals more easily than negatively charged materials [101]. Another study showed that zwitterionic‐based biomaterials can activate monocytes and dendritic cells, subsequently regulating macrophage polarization [102].

**FIGURE 9:**
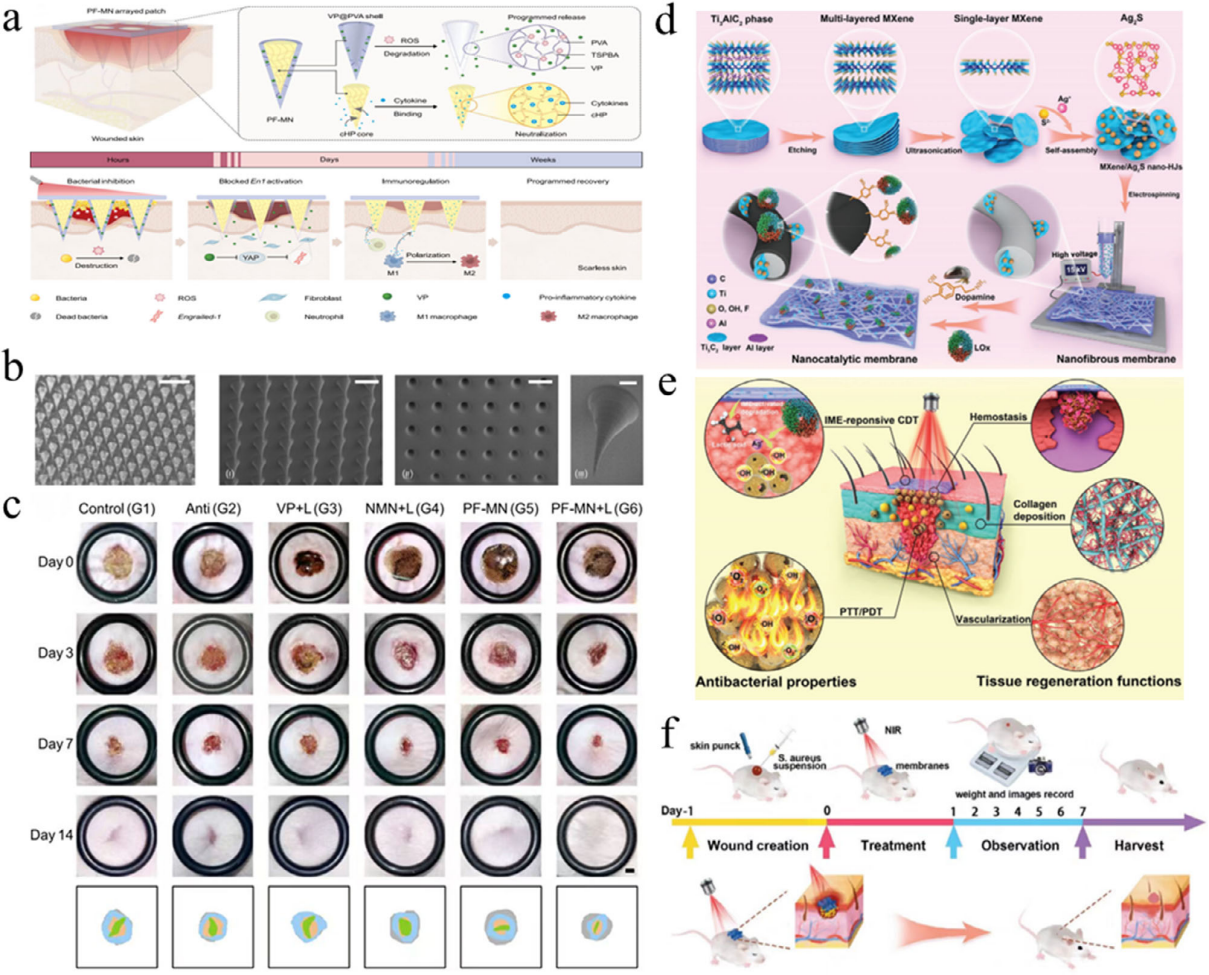
The application of engineered biomaterials in scarless wound healing. (a) The fabrication of PF‐MNs. (b) Photograph of the PF‐MN patch. (c) Photographs of wound healing [103]. Copyright 2023, Springer Nature. (d) Fabrication of the P‐MX/AS@LOx membrane. (e) Schematic diagram of the different treatment methods. (f) Schematic diagram of treatment approaches at different time points [104]. Copyright 2022, Wiley‐VCH.

The discovery of these physiological properties of biomaterials provides theoretical support for their use in immune modulation. By combining the immune modulation capabilities of biomaterials with good biocompatibility, bioactive scaffolds have been developed that can act as both regulators and delivery carriers. A programmable core‐shell structured MN array patch (PFMN) was developed (Figure 9a–c) [103]. The outer shell of the MN is made of a PVA layer containing the drug verteporfin (VP). VP is connected to PVA through an active oxygen responsive crosslinker (TSPBA). VP is not only a YAP inhibitor but also an effective antiscarring drug but also a photosensitizer. Under laser irradiation, ROS are produced to promote the degradation of the shell layer and further promote drug release. The core structure of the MN is crosslinked heparin (cHP), which can neutralize inflammatory factors in the wound environment and promote macrophage polarization toward the anti‐inflammatory phenotype. Unlike most MNs (polymeric MNs) that only control the release of therapy through encapsulation, PFMNs can dynamically regulate the wound immune microenvironment according to different healing stages and further dynamically adjust the degradation rate of the shell and the release profile of VP according to the inflammation status of the wound surface, enhancing personalized clinical treatment.

The degradation products of biomaterials in the body are equally important for tissue regeneration control. First, it is necessary to ensure that the degradation products are harmless. In addition, degradation products regulate tissue regeneration microenvironments by recruiting endogenous immune cells and stem cells and even induce the differentiation of stem cells in the body to affect tissue regeneration. An infection microenvironment (IME)‐activated nanocatalytic film composed of electrospun PLGA membrane‐biological heterojunction‐lactic acid oxidase (LOX) was developed (Figure 9d–f) [104]. The electrospun PLGA degrades in the acidic environment of the wound to produce lactic acid, and LOX decomposes lactic acid to produce a large amount of H_2_O_2_ as a reaction raw material. Subsequently, the biological heterojunction produces ·OH through a Fenton‐like reaction to enhance chemodynamic antibacterial action, achieving synergistic photothermal/photodynamic/metal ion antibacterial effects. This study developed a multimodal synergistic antibacterial intelligent system that combines environmental responsiveness and adaptability, providing guidance for the design of nanocatalytically active IME‐responsive materials.

The application of dynamic biomaterials in the field of tissue regeneration is constantly emerging [105]. The future development of biomaterials will focus on integrating independent and different dynamic responses into the same material system and achieving precise control of the system's programmed interactions [106]. Although these new methods have broad prospects, only a few methods have proven efficacy in vivo. Our goal is for research to prioritize the design and validation of novel techniques aimed at regulating the dynamic interactions of matrix properties, thereby fostering in situ tissue regeneration.

### 4.2 Signal Pathway Joint Control

In adult mammals, skin injury leads to the rapid proliferation of fibroblasts and deposition of ECM to form fibrotic scars for wound healing, resulting in the loss of functional tissue. Since Michael Harrison discovered that fetal wounds do not produce scars, researchers have studied the reasons why fetal wound repair does not lead to excessive fibrosis. It was later found that not only can fetuses resist scars, but also that the scars of elderly individuals are usually thinner than those of young people, and the same results have been observed in rodents. By exposing old mice to the blood of young mice, it was found that the scars formed after wound healing in old mice were thicker than those in the control group. Further experiments identified a possible factor causing this phenomenon: CXCL12, a gene encoding stromal cell‐derived factor 1 (SDF1), a protein. After removing SDF1, even young mice produced thinner scars [107], suggesting that inhibiting CXCL12 activity is a potential antiscarring approach.

Similar to fetal skin tissue, scientists have found that reindeer are an exception among adult mammals. Reindeer antlers regenerate annually and grow more than 1 cm per day, with a special skin called velvet covering the growing antlers. The velvet is pigmented, highly innervated, and vascularized, with a large number of embedded hair follicles (HFs) and sebaceous glands. Moreover, when the antlers are broken off, they can fully regenerate. Therefore, researchers have analyzed the molecular events that occur during the regeneration process of antlers to identify clues for scarless wound repair. Experiments have compared the differences between the wound repair processes of antlers and the back skin of reindeer to discover potential biological mechanisms involved [108]. The first difference is the immune environment. Antlers contain more resident immune cells than do back skin (accounting for 25% of the total cells), with CSF1R+ (colony stimulating factor 1 receptor) macrophages being enriched in the back skin. CSF1R activation usually leads to the activation and proinflammatory response of macrophages. Second, the most significant difference between the two sites was in fibroblasts, which are crucial for wound repair outcomes. Back skin fibroblasts uniquely express “proinflammatory” genes (CXCL1, CXCL3, and CCL2), while dFBs in antlers adopt an immunosuppressive phenotype, limiting leukocyte recruitment and accelerating immune resolution. In contrast, back skin fibroblasts maintain an activated myofibroblast‐like state, while antler skin fibroblasts exhibit stronger regenerative properties. By inducing the development of fibroblasts and matrix immune signals, it is possible to simulate the complete regeneration of skin at fetal and antler sites. This study proposes that the decoupling of fibroblast‐immune interactions is a promising method for reducing scarring.

In addition, cellular retinoic acid‐binding protein 1 (CRABP1) is reactivated in the dFBs of antlers. CRABP1 is a cellular retinoic acid‐binding protein that mainly binds and transports retinoic acid (retinoic acid) within cells and regulates the metabolism and function of intracellular RA. RA, as a checkpoint for the differentiation of CD201+ fibroblast progenitor cells into proinflammatory cells and myofibroblasts, plays a role in the generation of proinflammatory fibroblasts with excessive RA activity. Excessive activation of RA inhibits the transformation of proinflammatory fibroblasts into myofibroblasts, thus reducing scar formation [109].

The metabolism of another form of retinoic acid, vitamin A, has been found to upregulate the plasticity of stem cell lineages during the re‐epithelialization process of skin wounds. This effect depends on a small molecular metabolite of vitamin A, all‐trans retinoic acid (atRA), which can change the fate of mouse HFSCs, allowing stem cells to repair skin or grow hair. Interestingly, atRA cannot act alone; it interacts with BMP and WNT signaling molecules, affecting whether stem cells remain static or actively participate in hair regeneration [110]. Controlling the combination of vitamin A metabolism and related signaling pathways to control the differentiation route of stem cells at the wound site to achieve scarless wound repair is undoubtedly a novel and advantageous therapeutic method.

## 5 Summary

This review presents a detailed examination of the mechanisms behind scar formation and inhibition during wound healing, and it highlights the current advancements in therapeutic strategies aimed at minimizing scar tissue. Scar formation is a natural part of the healing process, typically characterized by the abnormal deposition of collagen in the skin, resulting in fibrotic tissue that differs in structure and function from normal skin [111]. While minor scars generally pose no significant problems, more severe scars, especially those resulting from large burns or traumatic wounds, can lead to severe functional and cosmetic impairments [112]. These include restrictions in movement, loss of skin appendages like hair follicles, and the inability to perform basic physiological functions like sweating, which can be distressing both physically and psychologically for patients. The review underscores the complexity of skin wound healing, which involves multiple cell types, the ECM, and immune responses. Unlike other tissues, skin regeneration is not merely a process of healing but also a process of remodeling, where a variety of cells, such as fibroblasts, keratinocytes, and endothelial cells, as well as factors like growth factors and cytokines, are involved in a highly coordinated manner. This intricate process leads to the formation of scar tissue, which lacks the full functionality of normal skin. The review concludes that current treatments are inadequate in addressing all aspects of scar inhibition. Most therapeutic approaches have focused on single targets, such as fibroblasts or collagen deposition, but these treatments often fall short of achieving full skin regeneration. As a result, future therapeutic strategies need to be more comprehensive, addressing multiple factors involved in the scar formation process, to achieve true tissue regeneration.

Looking ahead, there are several promising directions in the field of scar inhibition that hold significant potential. First, the need for multi‐target therapeutic strategies is paramount. Current treatments often focus on one factor in the healing process, such as the inhibition of collagen deposition or the suppression of inflammatory cytokines, but these approaches are not enough to fully restore skin to its normal, functional state. True skin regeneration, which would restore not only the skin's structure but also its functionality, will require therapies that address multiple biological pathways, including fibroblast activity, ECM remodeling, angiogenesis, and immune modulation. Recent advancements in understanding the complex biology of wound healing suggest that a more holistic, multi‐target approach is essential to overcoming the limitations of current therapies. Additionally, a major challenge in the field is the need for personalized treatments. Scar formation varies significantly among individuals due to genetic factors, the type of injury, and environmental influences, which makes it difficult to design a one‐size‐fits‐all treatment. Personalized medicine, based on individual genetic profiles, could help in tailoring specific therapies that are more likely to succeed for a given patient. With the increasing availability of genetic information and advancements in diagnostic tools, there is hope that personalized treatments could be developed to address the unique characteristics of each patient's wounds and scars. One of the most promising areas in scar inhibition is the potential of gene therapy and stem cell treatments. Gene therapy offers the possibility of directly manipulating the genetic pathways that lead to scar formation, potentially providing long‐term solutions. Similarly, stem cell therapies hold the promise of regenerating damaged tissues and promoting the healing of wounds with minimal scarring. However, translating these therapies from the laboratory to the clinic has been challenging. Issues such as the stability of gene therapy vectors, the risk of immune rejection of stem cells, and the potential for off‐target effects need to be addressed before these therapies can be widely adopted. Another major obstacle is the long‐term efficacy of current treatments. While some therapies may show short‐term benefits in scar reduction, their effectiveness may diminish over time, and side effects may emerge. This raises important questions about the long‐term sustainability of these treatments and their safety. Moreover, many of the current treatments do not address the full range of factors that contribute to scar formation, such as the interactions between the immune system and wound healing processes, making it essential to develop treatments that not only reduce visible scarring but also ensure the full restoration of skin function. The biocompatibility of materials used in scar inhibition treatments is another area that requires further exploration [113]. Treatments that involve implants, such as scaffolds or drug delivery systems, must be carefully designed to avoid immune rejection and other adverse reactions. The materials used must be both effective in promoting healing and non‐toxic over long periods. Researchers are exploring various biomaterials that can support tissue regeneration while also being compatible with the body's immune system.

Despite current challenges, the outlook for scar inhibition and wound healing therapies remains highly promising. The rapid advancements in several cutting‐edge biomedical fields, including gene editing, stem cell biology, biomaterials science, and nanomedicine are continuously reshaping the theoretical framework and technological strategies of scar treatment. Among these, gene editing technologies such as CRISPR‐Cas9 and base editors offer new opportunities for the precise regulation of key fibrotic signaling pathways, including TGF‐β/Smad, Wnt/β‐catenin, and Hippo‐YAP. Through localized or transient genomic modulation, it may become feasible to suppress pro‐fibrotic gene expression in dFBs or to reprogram scar‐resident myofibroblasts toward regenerative phenotypes. To facilitate clinical translation, the development of non‐viral delivery systems such as lipid nanoparticles and exosome‐based carriers with high transfection efficiency and low immunogenicity is urgently needed to ensure both safety and precision. Concurrently, stem cell‐based therapies have demonstrated significant potential in promoting tissue regeneration. Whether derived autologously or allogenically, MSCs, epidermal stem cells, and iPSCs not only differentiate into skin‐relevant cell types but also exert paracrine effects that modulate local immunity, enhance angiogenesis, and remodel the ECM. Future studies should prioritize the optimization of delivery methods (e.g., hydrogel encapsulation, scaffold seeding), long‐term safety assessments (e.g., tumorigenicity, immunogenicity), and the establishment of good manufacturing practice‐compliant production workflows. Nanotechnology provides valuable support in enhancing the precision and efficacy of scar therapies. Functionalized nanocarriers, such as pH‐ or ROS‐responsive nanoparticles, enable on‐demand drug release at the wound site, thereby minimizing systemic toxicity and improving pharmacokinetic profiles. Antifibrotic agents, including pirfenidone, decorin mimetics, and TGF‐β inhibitors, as well as stem cell‐derived exosomes can be incorporated into these platforms for multi‐target synergistic effects. Moreover, intelligent biomaterials such as bioactive hydrogels, self‐assembling peptides, and decellularized ECM scaffolds are being engineered to replicate the biomechanical and biochemical characteristics of native skin. These scaffolds not only serve as vehicles for cell or drug delivery but also actively regulate the wound microenvironment by modulating oxygen tension, inflammatory status, and mechanical stress. The integration of spatiotemporally controlled release systems allows for dynamic adaptation to different phases of wound healing, thereby favoring regenerative rather than fibrotic outcomes. To advance the clinical translation of emerging anti‐scar strategies such as cellular reprogramming, multiple critical aspects must be addressed to enhance scientific validity, regulatory compliance, and practical applicability. It is essential to establish animal models that closely mimic the biological features of human scarring. While rodent models such as mice and rats are cost‐effective and technically accessible, their skin structure differs significantly from humans and often fails to reproduce the complex pathophysiology of hypertrophic or keloid scarring. Therefore, large animal models such as pigs and rabbits with skin anatomy, healing kinetics, and immune responses more comparable to those of humans are urgently needed to provide more reliable data on therapeutic efficacy and safety. Furthermore, standardized and reproducible efficacy evaluation systems and biosafety criteria are imperative for clinical translation. At present, universally accepted definitions for “scar improvement” or “regenerative healing” remain lacking, making inter‐study comparisons challenging. It is recommended that comprehensive evaluation protocols incorporate quantitative metrics such as scar volume, thickness, collagen alignment, vascularization, and appendage regeneration, alongside imaging, biomarker analysis, and histopathological assessment. Safety evaluations should address not only conventional concerns such as tumorigenicity and immunotoxicity, but also local inflammatory responses, degradation byproducts, and long‐term biocompatibility of implanted materials.

In summary, the field of scar inhibition is increasingly characterized by multidisciplinary convergence. The integration of gene editing, stem cell therapy, advanced biomaterials, nanotechnology, and personalized medicine holds the potential to overcome current therapeutic bottlenecks. With continued investment in both basic and translational research, as well as the implementation of large‐scale clinical trials, future scar therapies are expected to move beyond cosmetic improvements and achieve comprehensive structural and functional skin regeneration.

## Conflicts of Interest

Xiaoyuan Ji is a member of the *Exploration* editorial board, and he was not involved in the handling or peer review process of this manuscript. The other authors declare no conflicts of interest.

## Data Availability

No data was used for the research described in the article.
